# Single‐cell RNA‐seq reveals fate determination control of an individual fibre cell initiation in cotton (*Gossypium hirsutum*)

**DOI:** 10.1111/pbi.13918

**Published:** 2022-10-02

**Authors:** Yuan Qin, Mengling Sun, Weiwen Li, Mingqi Xu, Lei Shao, Yuqi Liu, Guannan Zhao, Zhenping Liu, Zhongping Xu, Jiaqi You, Zhengxiu Ye, Jiawen Xu, Xiyan Yang, Maojun Wang, Keith Lindsey, Xianlong Zhang, Lili Tu

**Affiliations:** ^1^ National Key Laboratory of Crop Genetic Improvement, Hubei Hongshan Laboratory Huazhong Agricultural University Wuhan Hubei Province China; ^2^ Department of Biosciences Durham University Durham UK

**Keywords:** cotton fibre initiation, single cell transcriptomic atlas, *Gossypium hirsutum*, regulatory network, cell fate determination

## Abstract

Cotton fibre is a unicellular seed trichome, and lint fibre initials per seed as a factor determines fibre yield. However, the mechanisms controlling fibre initiation from ovule epidermis are not understood well enough. Here, with single‐cell RNA sequencing (scRNA‐seq), a total of 14 535 cells were identified from cotton ovule outer integument of Xu142_LF line at four developmental stages (1.5, 1, 0.5 days before anthesis and the day of anthesis). Three major cell types, fibre, non‐fibre epidermis and outer pigment layer were identified and then verified by RNA *in situ* hybridization. A comparative analysis on scRNA‐seq data between Xu142 and its fibreless mutant Xu142 *fl* further confirmed fibre cluster definition. The developmental trajectory of fibre cell was reconstructed, and fibre cell was identified differentiated at 1 day before anthesis. Gene regulatory networks at four stages revealed the spatiotemporal pattern of core transcription factors, and *MYB25‐like* and *HOX3* were demonstrated played key roles as commanders in fibre differentiation and tip‐biased diffuse growth respectively. A model for early development of a single fibre cell was proposed here, which sheds light on further deciphering mechanism of plant trichome and the improvement of cotton fibre yield.

## Graphical abstract

With scRNA‐seq, cotton fibre cell was identified differentiated at −1 DPA. It further refines the spatiotemporal patterns of two command genes, MYB25‐like and HOX3, who determine fibre differentiation and tip‐biased diffuse growth respectively.

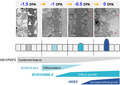

## Introduction

Cotton is an important cash crop worldwide, and supplies the largest proportion of natural fibre to textile industry. Cotton fibres are unicellular, and initiate from ovule epidermis, making it a good model for studying mechanisms of cell fate determination. There are two types of cotton fibre according to mature fibre length: lint and fuzz. The initiation of lint (long) fibre proceeds from the day post‐anthesis (0 DPA) to 3 DPA, with the initiation of fuzz (short) fibre typically commencing afterwards. The number of lint fibre initials per seed as a factor determines fibre yield, while lint initiation is typically from 25% of all epidermal cells (Stewart, 1975). Therefore, exploring the mechanisms and analysing key factors and networks that regulate lint fibre cell fate, can provide a theoretical basis for the genetic improvement of fibre yield, so as to help improve the economic benefits of cotton planting.

Numerous studies have investigated the mechanisms of fibre development by means of expression profiling. Since early 21st century, 14 highly expressed cDNAs were identified in cotton fibre using cDNA arrays (Li *et al*., 2002), after that several expression profiles have been performed on various stages of fibre development (Gou *et al*., 2007; Shi *et al*., 2006), between different cotton species (Tu *et al*., 2007), during domestication (Hovav *et al*., 2008; Rapp *et al*., 2010), between normal cotton and fibre‐related mutants (Wu *et al*., 2006). A few transcription factor (TF) genes, such as R2R3‐type MYB TFs *GhMYB109* (Pu *et al*., 2008), *GhMYB25* (Machado *et al*., 2009), *GhMYB25‐like* (Walford *et al*., 2011; Wan *et al*., 2016), and HD‐ZIP family TFs *GhHD‐1* (Walford *et al*., 2012), *GhHOX3* (Shan *et al*., 2014) have been verified positively regulating lint fibre initiation. A model has been proposed in which no lint fibre will initiate if the combined expression levels of *MYB25‐like_At* and *MYB25‐like_Dt* are below a critical threshold level at 0 DPA (Zhu *et al*., 2018). In addition, suppression of sucrose synthase activity by at least 70% in the ovule epidermis (Ruan *et al*., 2003), or of a vacuolar invertase gene *GhVIN1* (Wang *et al*., 2014), led to a fibreless phenotype. Furthermore, naked seeds were produced when ovules were cultured with adding no indoleacetic acid (IAA) (Zeng *et al*., 2019) or excess IAA transport inhibitor (Zhang *et al*., 2011; Zhang *et al*., 2017) or with high concentration of zeatin (ZT), a kind of cytokinin (Zeng *et al*., 2019). Recently, some review papers have summarized that fibre initiation was affected by complex cross‐talk among MYB MIXTA‐like TFs, sugar signals and plant hormones (Huang *et al*., 2021; Tian and Zhang, 2021; Wang *et al*., 2021a). Despite these studies, when did the members in the complex start work, whether they take action only in fibre cells or also in adjacent cells during the continuous fibre initiation process, were partly ambiguous and needs more details.

Now, single‐cell RNA sequencing (scRNA‐seq) has been developed and brings unprecedented opportunities to the field of plant research (Denyer and Timmermans, 2022; Mo and Jiao, 2022; Ryu *et al*., 2021; Seyfferth *et al*., 2021; Shaw *et al*., 2021). The first effective high‐throughput scRNA‐seq in plants exploited single‐cell transcriptome sequencing of *Arabidopsis* root tissue protoplasts (Ryu *et al*., 2019). Subsequently, a series of developmental processes in *Arabidopsis* had been explored at single cell resolution, such as the development of root tips (Denyer *et al*., 2019; Jean‐Baptiste *et al*., 2019; Shahan *et al*., 2022; Wendrich *et al*., 2020; Zhang *et al*., 2019), lateral root (Gala *et al*., 2021), vegetative shoot apex (Zhang *et al*., 2021b), stomatal cell lineage (Liu *et al*., 2020), developing leaf (Kim *et al*., 2021; Liu *et al*., 2022a; Lopez‐Anido *et al*., 2021; Tenorio Berrio *et al*., 2022) and vein pattern in the cotyledons (Liu *et al*., 2022b). At the same time, scRNA‐seq has also been widely used in other plants, including rice (Liu *et al*., 2021b; Wang *et al*., 2021b; Zhang *et al*., 2021a; Zong *et al*., 2022), corn (Li *et al*., 2022; Ortiz‐Ramirez *et al*., 2021; Satterlee *et al*., 2020; Sun *et al*., 2022; Xu *et al*., 2021), peanut (Liu *et al*., 2021a), tea plant (Wang *et al*., 2022), tomato (Omary *et al*., 2022) and poplar (Chen *et al*., 2021; Li *et al*., 2021; Xie *et al*., 2022). These studies provide new insights into heterogeneity of gene expression between different cell types, and molecular trajectory of cell differentiation during development.

To further decipher the detailed gene regulatory network in fibre initiation, we performed scRNA‐seq on cotton ovules. The developmental trajectory starting from early differentiated fibre cell was reconstructed. All the results can interactively be mined on the web, which is freely available at http://cotton.hzau.edu.cn/CLC/. The single cell resolution transcriptomes provide a valuable resource, and give us a deep understanding on the elaborate cotton fibre initiation process.

## Results

### Fibre cells begin to protrude at 0.5 days before anthesis

**Figure 1:**
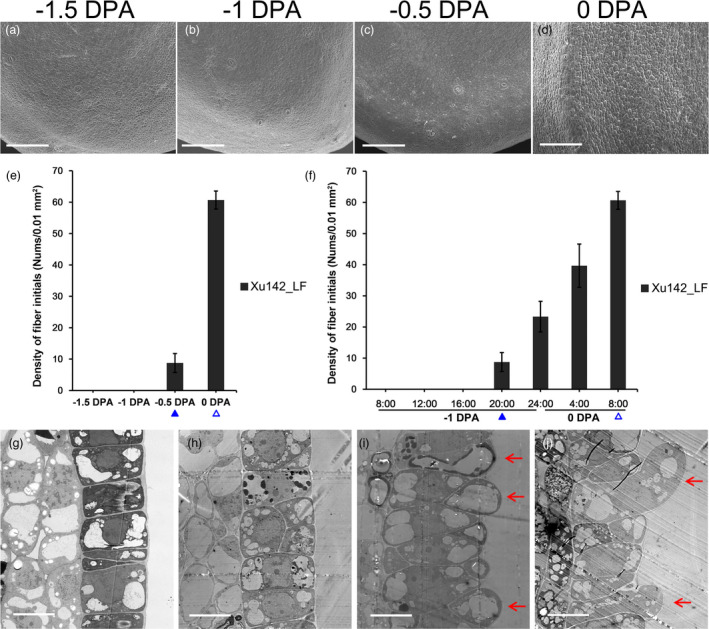
The phenotype of ovule epidermis of Xu142_LF line during fibre initiation. (a–d) Xu142_LF ovule epidermis observed by scanning electron microscopy (SEM) at −1.5, −1, −0.5 and 0 DPA respectively. (e) Statistics of fibre initials number on ovule epidermis per unit area (100 × 100 μm^2^) from (a–d). (f) Statistics of fibre initials number on ovule epidermis per unit area (100 × 100 μm^2^) from Figure S1. The blue solid triangles and hollow triangles marked stages in (e) and (f) mean the same developmental stage respectively. (g–j) Ovule epidermal cells observed by transmission electron microscope (TEM) at −1.5, −1, −0.5 and 0 DPA respectively. Red arrows in (i) and (j) point to fibre cells. Bars: 100 μm (a‐d); 10 μm (g–j).

The phenotype of cotton Xu142_LF line (seed index 10–12 g, lint index 6–7 g and lint percentage 35%–38%) has been described in our previous work (Hu *et al*., 2018). A more detailed morphological observations on Xu142_LF ovules were performed at −1.5, −1, −0.5 and 0 DPA respectively (Figure 1). By SEM, no fibre cell protrusion detected on ovule epidermis at −1.5 DPA (Figure 1a) and −1 DPA (Figure 1b), while at −0.5 DPA can be detected (Figure 1c), and protrusions number increased at 0 DPA (Figure 1d,e).

To further investigate the dynamic process of fibre initiation, samples were taken every 4 h (h) before and after fibre cell protrusion (from −1 to 0 DPA, Figure S1). No epidermal cell protruded at 08 : 00, 12 : 00 and 16 : 00 of −1 DPA. At 20 : 00, fibre cells began to protrude. At 24 : 00, the number of fibre initials increased. At 4 : 00 of 0 DPA, fibre initials grew larger, similar in size to those at 8 : 00 of 0 DPA (Figure S1). From 20 : 00 (−0.5 DPA), the number of fibre initials shows an increasing trend (Figure 1f).

Ovule epidermis at those four stages were also observed by TEM. Consistent with SEM observations, epidermal cells did not protrude at −1.5 and −1 DPA (Figure 1g,h), but began to protrude at −0.5 DPA (Figure 1i) and continued to expand at 0 DPA (Figure 1j). Considering lint fibre cell start protruding at −0.5 DPA, their fate is likely to be determined as early as −1 DPA, or −1.5 DPA, or possibly earlier.

### Fibre cell cluster is identified from cotton ovule outer integument by scRNA‐seq

To explore the molecular mechanism determining fibre cell fate, we performed scRNA‐seq on Xu142_LF ovules. Ovule samples collected at −1.5, −1, −0.5 and 0 DPA were used for protoplast isolating (Figure S2a,b). About 20 000 protoplasts were initially loaded onto the 10× Genomics platform. After separation, RNA from individual protoplasts were extracted for library construction followed by high‐throughput sequencing (Figure S2c). As shown in longitudinal section of ovules after enzymolysis, only cells from ovule outer integument were released (Figure S2d–g).

In Xu142_LF 0 DPA sample (LF_0d), a total of 3679 cells with 50 753 genes were detected (Table S1). After a strict gene/cell filtering process (Appendix S1), LF_0d sample obtained 35 169 gene transcripts with high reliability across 1703 cells. This filtration was performed on other samples one by one, then high‐quality gene‐cell matrices were obtained. Overall, 738–2045 filtered cells per sample were obtained (Table S1).

**Figure 2:**
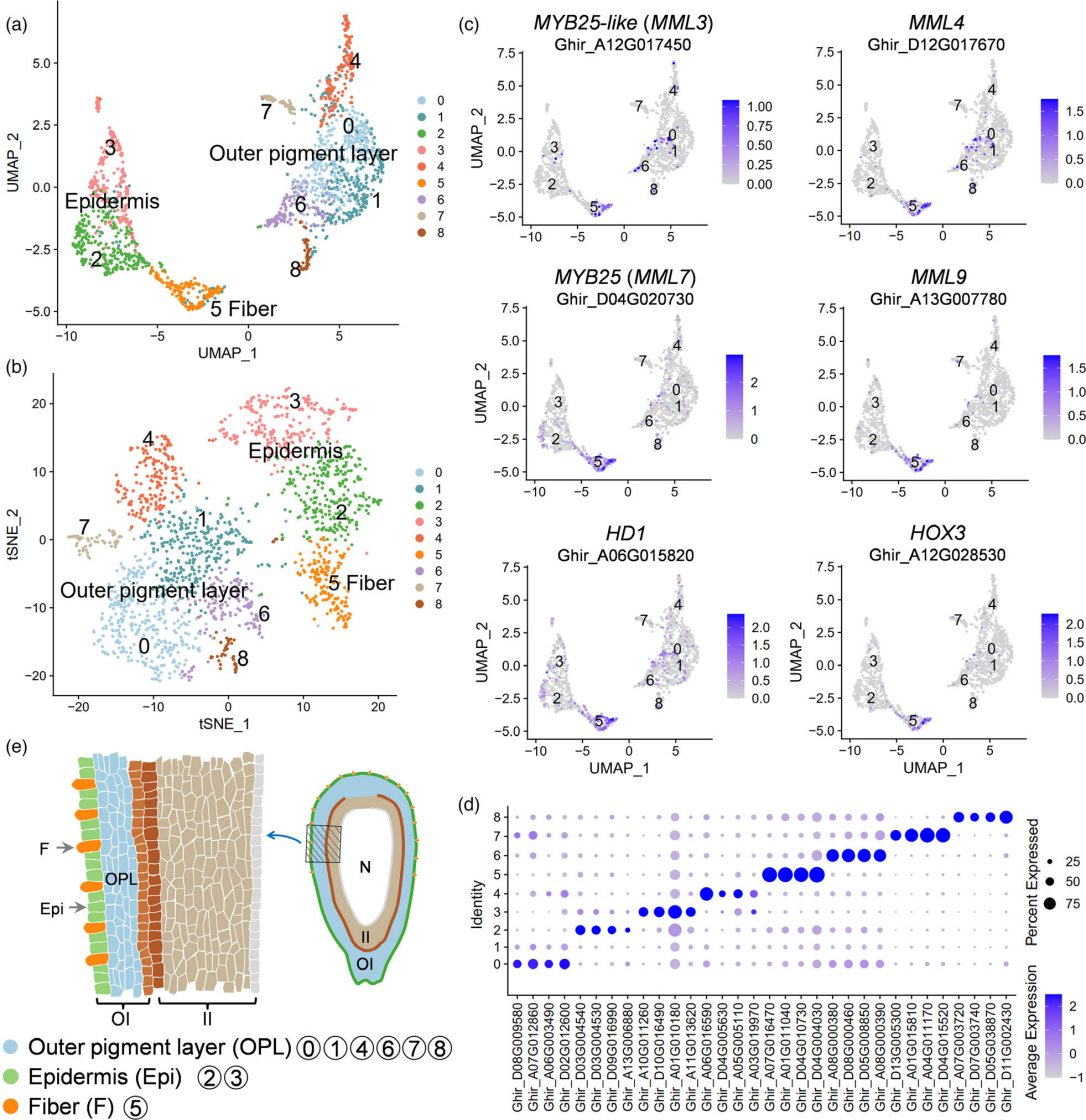
Cluster annotation of single‐cell transcriptomes from cotton ovule outer integument. (a) UMAP visualization of putative clusters from 1703 cells in cotton ovule outer integument of LF_0d sample. Each dot denotes a single cell. Colours denote corresponding cell clusters. Resolution was 0.8. (b) *t*‐SNE projection plot showing major clusters of the 1703 individual cell transcriptomes of LF_0d sample. (c) UMAP projection plots showing transcript accumulation for known fibre markers in individual cells. Colour intensity indicates the relative transcript level for the indicated gene in each cell. (d) Expression pattern of top four genes enriched in each cluster of LF_0d. Dot diameter, proportion of cluster cells expressing a given gene; colour, average expression across cells in that cluster. (e) Schematic diagram of longitudinal section of 0 DPA cotton ovule showing spatial distribution of cell clusters in ovule outer integument. The left part is a magnified view of the shaded part on the right. F, fibre; Epi, epidermis; OPL, outer pigment layer; OI, outer integument layer; II, inner integument layer; N, nucellus.

To examine the robustness of the scRNA‐seq results, LF_0d scRNA‐seq data were compared with 0 DPA ovule outer integument bulk RNA‐seq data (Hu *et al*., 2018). The correlation coefficient (R) was 0.63 with *P* < 2.2e‐16, showing a very significant correlation between them (Figure S3a). There were 3974 protoplasting‐induced differentially expressed genes (DEGs) identified, with 2233 DEGs up‐regulated and 1741 down‐regulated (Table S2). GO analysis suggested that the up‐regulated genes were involved in ‘response to stress and stimulus’ and ‘regulation of cell death’, among others (Figure S3b); the down‐regulated genes were mainly involved in ‘primary metabolic process’ and ‘biosynthetic process’ (Figure S3c). Next, UMAP and *t*‐SNE algorithm were used to visualize and explore LF_0d dataset after linear dimensional reduction. Unsupervised analyses grouped 1703 cells into nine clusters (Figure 2a,b). Similarly, nine clusters were also observed after 3974 DEGs in response to protoplasting were removed (Figure S3d), and clustered cells were almost kept in the same cell types as before (Figure S3e). This suggested that cell wall enzymolysis had only a minor effect on cell clustering, the same as reported in rice root (Liu *et al*., 2021b).

**Figure 3:**
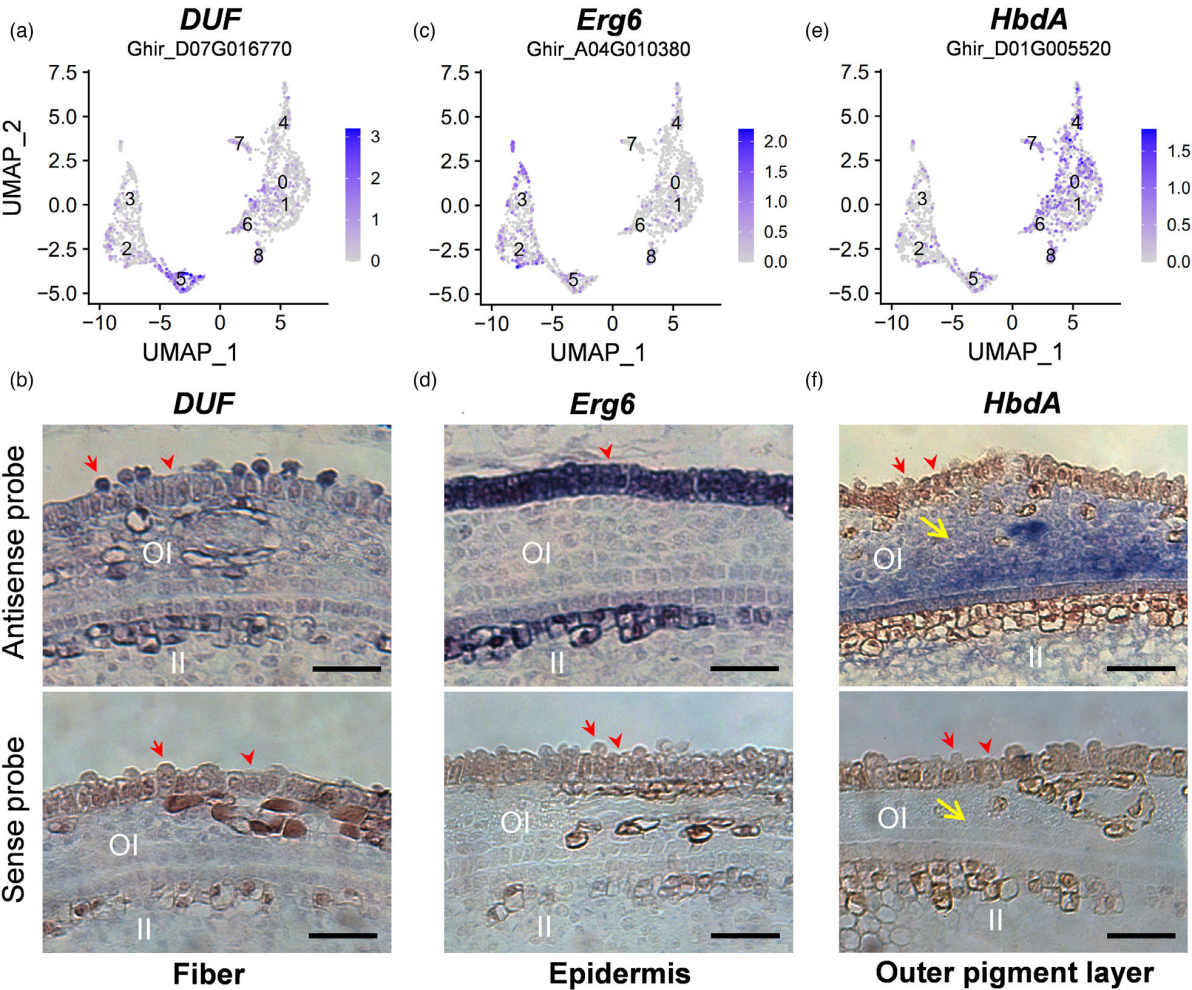
RNA *in situ* hybridization of cell‐type representative marker genes. (a) UMAP projection plots showing transcript accumulation for *DUF* (*Ghir_D07G016770*, function unknown), a novel fibre marker gene from cluster 5. (b) RNA *in situ* hybridization of *DUF* with the sense probe as a negative control. (c) Expression of non‐fibre epidermis novel marker gene *Erg6* (*Ghir_A04G010380*, a methyltransferase encoding gene) in cluster 2 and 3. (d) RNA *in situ* hybridization of epidermis marker (*Erg6*) with the sense probe as a negative control. (e) UMAP projection plots showing transcript accumulation for outer pigment layer novel marker gene *HbdA* (*Ghir_D01G005520*, 3‐hydroxyacyl‐CoA dehydrogenase family protein) in cluster 0, 1, 4, 6, 7 and 8. (f) RNA *in situ* hybridization of *HbdA* with the sense probe as a negative control. The hybridization signals of these marker genes in whole cotton ovules were shown in Figure S5. Sections (10 μm) from Xu142_LF 0 DPA ovules were used for *in situ* hybridization. OI, outer integument; II, inner integument. Red arrows indicate fibre cells, and red arrowheads indicate non‐fibre epidermal cells. Yellow arrows indicate outer pigment layer. Scale bars, 50 μm.

For cell cluster definition, accumulation of reported fibre gene transcripts in single‐cell populations were analysed (Figure 2c, Figure S4a). The fibre genes included *MYB25‐like* (Walford *et al*., 2011; Wan *et al*., 2016), *MYB25* (Machado *et al*., 2009), *MML4* (Wu *et al*., 2018), *MML9* (Bedon *et al*., 2014), *HD1* (Walford *et al*., 2012) and *HOX3* (Shan *et al*., 2014). All these genes tended to be highly or preferentially expressed in cluster 5 (Figure 2c, Figure S4b), indicated that cluster 5 may be fibre cell. To further enable cell type assignment to particular clusters, a series of enriched genes for each cluster were identified (Figure 2d, Figure S4c, Table S3). With RNA *in situ* hybridization of the representative genes, all cell clusters from cotton ovule outer integument can be defined into three major types: fibre cell, non‐fibre epidermis and outer pigment layer (Figures 2e and 3). For example, *DUF* (*Ghir_D07G016770*, gene function unknown) was preferentially expressed in fibre cells, *Erg6* (*Ghir_A04G010380*, a methyltransferase encoding gene) in non‐fibre epidermis and *HbdA* (*Ghir_D01G005520*, 3‐hydroxyacyl‐CoA dehydrogenase) in outer pigment layer (Figure 3, Figure S5).

### Fibre cell definition is further proved by scRNA‐seq of *Xu142 fl* fibreless mutant

**Figure 4:**
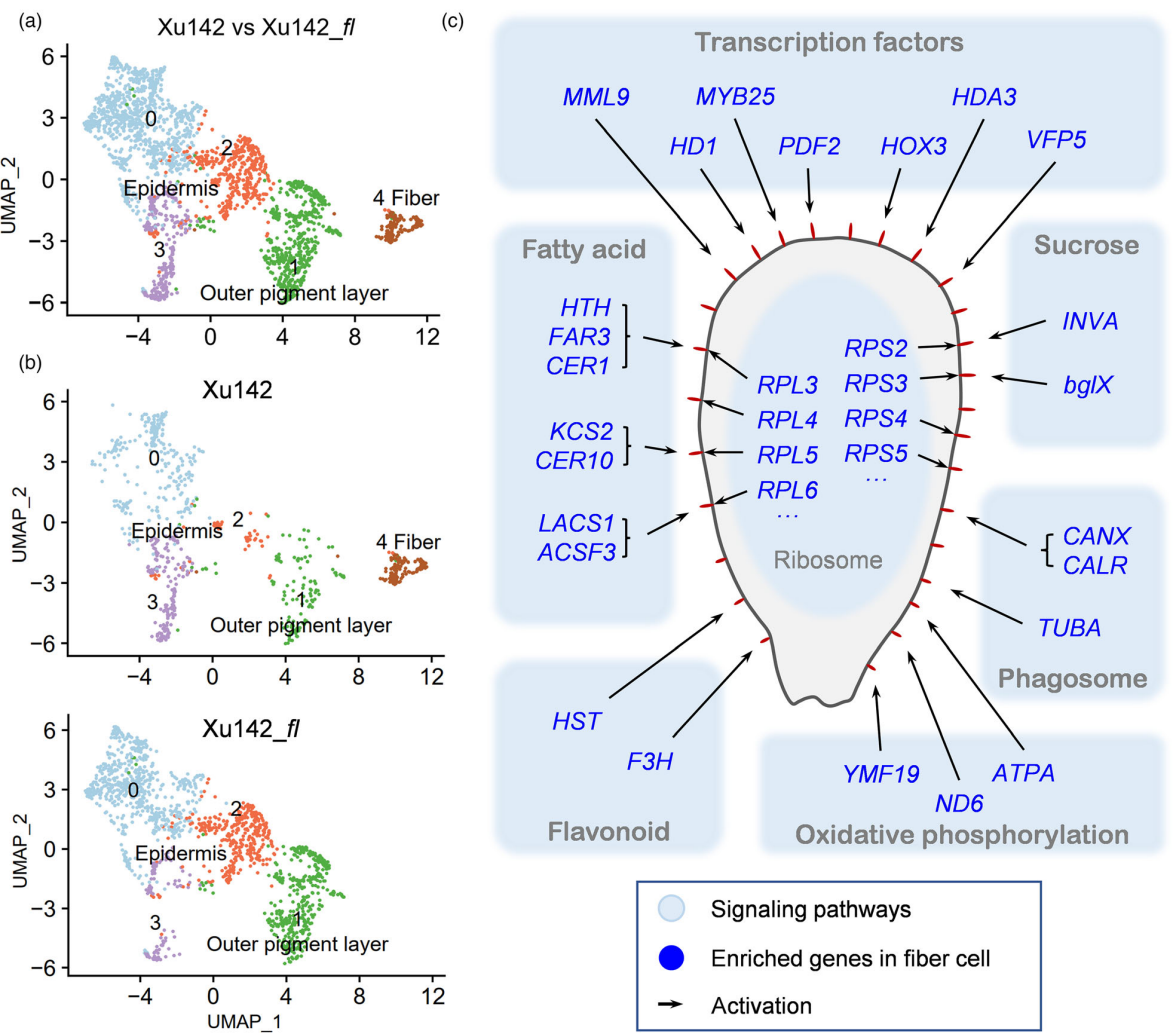
Clarifying of fibre cell cluster and its enriched pathways. (a) UMAP visualization of putative clusters in cotton ovule outer integument of Xu142 versus Xu142 *fl* sample. Each dot denotes a single cell. Colours denote corresponding cell clusters. Resolution was 0.2. (b) UMAP visualization of putative clusters which shown separately according to Xu142 and Xu142 *fl*. (c) Schematic diagram showing the main pathways and regulatory factors active in 0 DPA fibre cells. The red protrusions on the ovule epidermis represent protruded fibre cells.

Single‐cell transcriptomes were generated from 0 DPA ovule protoplasts of wild type cultivar Xu142 and its fibreless mutant Xu142 *fl*. Clustering these two samples together generated five clusters (Figure 4a, Table S3), which were assigned to three major cell types using *in situ* hybridization‐verified genes: fibre (cluster 4), non‐fibre epidermis (cluster 0, 2 and 3), outer pigment layer (cluster 1, Figure 4a, Figure S6a). When comparing the clustering results between Xu142 and Xu142 *fl*, the fibre cell cluster (cluster 4) was found to exist only in Xu142, not in Xu142 *fl* (Figure 4b, Table S4). The expression distribution of three fibre marker genes (*MYB25*, *MML9* and *HOX3*) also showed expressing in Xu142, not in Xu142 *fl* (Figure S6b). These again proved that the fibre cell definition was reliable.

### ‘Ribosome’ pathway is enriched in 0 DPA fibre cells

To compare the fibre cluster enriched genes between LF_0d and Xu142_0d, a clustering analysis on Xu142_0d sample was performed. The 738 high‐quality cells from Xu142_0d clustered into 6 clusters. Cluster 2 was identified as fibre cell with expression pattern analysis of fibre markers (*MYB25* and *MML9*, Figure S6c,d), and 984 genes were identified enriched in fibres (Table S3). When comparing these 984 genes with the 517 genes enriched in LF_0d fibres (Table S3), it was found that 474 genes overlapped, which means a similarity of gene expression between them (Figure S6e).

To explore the pathways that are active in fibre cells, KEGG enrichment analysis was performed on the 517 genes enriched in LF_0d fibres. These genes were involved in 38 different signalling pathways (Table S5). Among them, 312 genes were involved in the most significant pathway ‘Ribosome’ (Table S5), which indicated that a major activity in 0 DPA fibres was ‘peptide biosynthetic process’. It was consistent with the characteristics of expanding fibre initial cells, that is, more abundant transcripts involved in protein synthesis to meet the high demand for new cell wall and membrane components (Wu *et al*., 2007). The remaining genes were enriched in several pathways such as ‘Fatty acid’, ‘Flavonoid’, ‘Oxidative phosphorylation’, ‘Sucrose’ and ‘Phagosome’ (Figure 4c, Table S5). Besides, some TFs were identified from the 517 genes, including *MML9*, *MYB25*, *HD1*, *PDF2*, *HOX3*, *HDA3* and *VFP5* (Figure 4c). These TFs should play positive roles in 0 DPA fibre development, as some of them have been experimentally verified (Machado *et al*., 2009; Shan *et al*., 2014; Walford *et al*., 2012).

### Fibre cell was differentiated at −1 DPA

**Figure 5:**
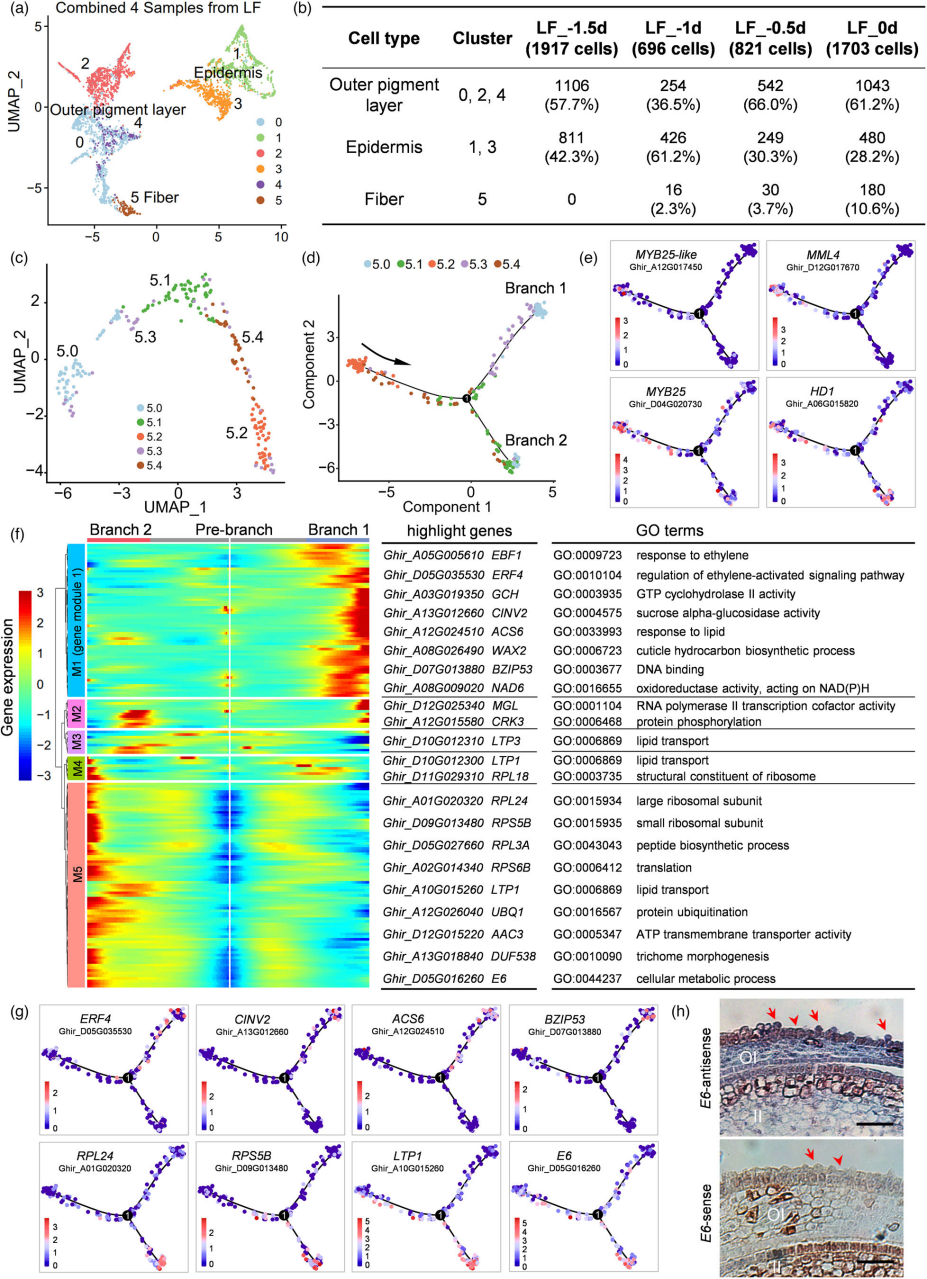
Developmental trajectory of fibre cells. (a) UMAP visualization of putative clusters of the combined sample. The scRNA‐seq data of −1.5, −1, −0.5 and 0 DPA samples were combined for clustering. Each dot denotes a single cell. Colours denote corresponding cell clusters. Resolution was 0.2. (b) Cell number of each type identified from the combined sample, which shown at −1.5, −1, −0.5 and 0 DPA respectively. (c) Re‐clustering of all fibre cells identified in the combined sample. (d) A pseudotime trajectory showing fibre cells development. The developmental branch locations of 5 sub‐clusters. Different colours represent the cells from each sub‐cluster. (e) Expression patterns of fibre marker genes (*MYB25‐like*, *MML4*, *MYB25* and *HD1*). The colours represent expression levels of these genes in individual cells. (f) Heat map displays the 167 branch‐dependent genes with qval <0.001. Each row represents one gene. These genes were clustered into 5 modules with distinct expression patterns. Different colours represent the gene expression level. The representative genes of each module are shown in the middle panel. The gene ontology (GO) terms for each module are shown on the right panel. (g) Representative marker genes (*ERF4*, *CINV2*, *ACS6* and *BZIP53*) expressed in branch 1 (upper panel), and expression of representative marker genes (*RPL24*, *RPS5B*, *LTP1* and *E6*) in branch 2 (lower panel). The colour bar indicates relative expression levels. (h) RNA *in situ* hybridization of *E6* gene (*Ghir_D05G016260*) in LF_0d ovules with the sense probe as a negative control. OI, outer integument; II, inner integument. Red arrows indicate fibre cells, and red arrowheads indicate non‐fibre epidermal cells. Scale bars, 50 μm.

In order to clarify the dynamic changes of gene expression accompanying fibre cell initiation, the single‐cell transcriptomes in Xu142_LF across four stages (−1.5, −1, −0.5 and 0 DPA) were combined. After unsupervised clustering, totally 5137 cells were clustered into six clusters (Figure 5a), and genes enriched in each cluster were identified (Table S3). Based on the well‐defined cell types in LF_0d and cell barcode mapping (Table S6), the combined sample can be defined as fibre (cluster 5), epidermis (cluster 1, 3) and outer pigment layer (cluster 0, 2 and 4; Figure 5a). The expression pattern of fibre marker genes (*MYB25*, *MML9* and *HD1*) further verified that cluster 5 was fibre cell cluster (Figure S7a).

When displaying the clustering result separately at four stages (Figure S7b–e), no fibre cell was identified at −1.5 DPA (Figure S7b), while the next three stages contained fibre cluster (Figure S7c–e). The expression pattern of *MYB25* and *MML9* also showed that they did not express at −1.5 DPA, but expressed at next three stages (Figure S7f,g). According to previous phenotypic observation, −0.5 DPA was the time point showing visible fibre initials (Figure 1i). Here, the transcriptome data suggested that fibre cells were differentiated at −1 DPA (Figure S7c), although they were morphologically undistinguishable to its neighbouring cells at this stage (Figure 1h). Since then, the number of fibre cell (cluster 5) increases stepwise along development (Figure 5b).

Next, the developmental trajectory of fibre cells was explored. In total, 226 fibre cells (16 cells from LF_‐1d, 30 cells from LF_‐0.5d and 180 cells from LF_0d, Figure 5b) were selected for re‐clustering. There were five sub‐clusters identified (Figure 5c). When displaying the re‐clustering result separately (Figure S7h), it was found that −1 DPA fibre cells were mainly located to sub‐cluster 5.2. Then, pseudotime analysis on these fibre cells built a developmental trajectory (Figure 5d). The starting of the trajectory was specified at the left branch, as sub‐cluster 5.2 cells (early fibre cells) were located on this branch. There was a node in the trajectory, with branching into two directions (Figure 5d). The MYB‐MIXTA‐like TFs, such as *MYB25‐like*, *MML4* and *MYB25*, were prominently highly expressed at the beginning branch (Figure 5e), in agreement with their key roles in fibre cell differentiation (Machado *et al*., 2009; Walford *et al*., 2011; Wu *et al*., 2018). The homeodomain leucine zipper transcription factor, *HD1* (Walford *et al*., 2012), was expressed almost at all the branches (Figure 5e), suggesting that *HD1* gene has a broader temporal pattern during fibre initiation, distinct from MYB‐MIXTA‐like TFs.

It is known that once early fibre cell differentiated, they will undergo a process of diffuse growth then transformed into tip‐biased diffuse growth (Qin and Zhu, 2011; Yu *et al*., 2019). To further define the cell types on branch 1 and branch 2, a total of 167 genes were firstly identified as most significantly related to branching (qval < 0.001, Table S7). A heat map containing these 167 genes was produced and they were sorted into five modules (Figure 5f). Among them, 59 genes in gene module 1 (M1) are predominantly expressed in branch 1 cells. GO results show that they are mainly enriched in ‘regulation of ethylene‐activated signalling pathway’, ‘sucrose alpha‐glucosidase activity’, ‘cuticle hydrocarbon biosynthetic process’ and so on (Figure 5f, Table S8). These GO terms were reported mainly enriched in fibre elongation (Shi *et al*., 2006). In details, multiple genes involved in ethylene signalling, such as ethylene responsive element binding factor (*ERF4*, *ERF9* and *ERF11*), and 1‐aminocyclopropane‐1‐carboxylic acid (ACC) synthase gene (*ACS6*) (Shi *et al*., 2006) were all prominently expressed in branch 1 cells (Figure 5g, Table S7). There are 79 genes in M5, which are predominantly expressed in branch 2 cells. They are mainly enriched in ‘ribosome’, ‘translation’, ‘lipid transport’ and ‘ATP transmembrane transporter activity’ (Figure 5f, Table S8), these GO terms were reported highly enriched in diffuse growing fibre cells (Wu *et al*., 2007). The genes encoding large and small ribosomal subunit protein (*RPL24*, *RPS5B*, *RPL3A* and *RPS6B*) and lipid transfer protein (*LTP1*) (Wu *et al*., 2006) were highly enriched in branch 2 (Figure 5g, Table S7). Moreover, an E6 protein encoding gene (*Ghir_D05G016260*), which plays a role in diffuse growing fibre cell (Ji *et al*., 2003), was identified and verified by *in situ* hybridization (Figure 5g,h).

As described above, fibre initiation can be subdivided into several processes. Precursor fibre cell have not differentiated at −1.5 DPA (Figure S7b), and then differentiated into fibre cell at −1 DPA (Figure S7c and Figure 5b) and start protruding by diffuse growth at −0.5 DPA (Figure 1i), then transformed into tip‐biased diffuse growth from 0 DPA (Figure 1j).

### The highly interconnected gene regulatory networks coordinate fibre cell initiation

The results above have shown that these four stages (−1.5, −1, −0.5 and 0 DPA) represented distinct processes of fibre initiation. To explore gene regulatory networks in each process, clustering analysis were performed on four samples individually (Figure S8a). By analysing transcript accumulation of fibre marker gene *MML4* and the *in situ* hybridization‐verified genes (*DUF*, *Erg6* and *HbdA*, Figure S8b), the clusters in each sample can be defined into fibre, epidermis and outer pigment layer (Figure S8a). Then, genes enriched in each cluster were identified (Table S9), and WGCNA was performed on four samples respectively. Several distinct gene modules (labelled by different colours) were identified as shown in the dendrogram (Figure S9, Table S10). The modules containing genes enriched in fibre cells (−1, −0.5 and 0 DPA) or cells that may differentiate into fibre (−1.5 DPA) were selected for co‐expression network construction (Figures S9 and S10, Table S11).

Among these four networks, some core components shared between adjacent stages, while some were specific at one stage. For example, the PROTODERMAL FACTOR1 (*PDF1*) gene, whose silencing caused retardation of fibre initiation (Deng *et al*., 2012), was identified as a core network component both at −1.5, −0.5 and 0 DPA (Figure S10). Two genes coding lipid transfer protein (*LTP3* and *LTP6*), which can transport metabolites around the cell for their membrane biosynthesis (Wu *et al*., 2006; Wu *et al*., 2007), were the core components at both −1.5 and −1 DPA network. At −0.5 DPA network, AMP‐dependent synthetase and ligase (*LACS1*), ABC‐2 type transporter (*ABCG13*), Ribosomal protein (*RPS29A*) and a transcription factor *MYB25* were located in the core (Figure S10). At 0 DPA, the core network components including some function unknown genes, such as *Ghir_D08G021100*, *HUTL*, *Era* and others.

**Figure 6:**
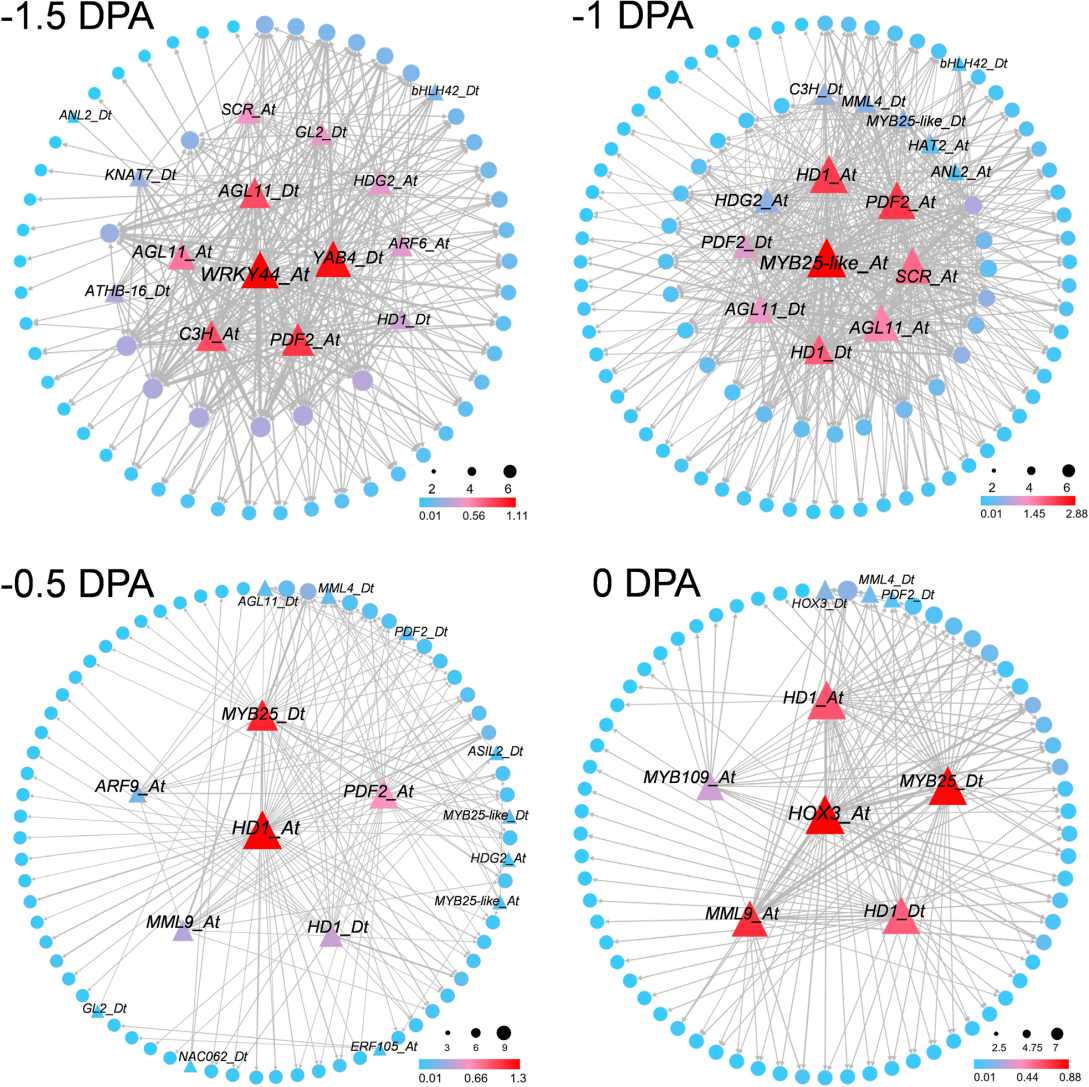
Transcription factors (TFs) regulatory network predicts key regulators in the four distinctive developmental processes during fibre initiation. A total of 15, 15, 16 and 9 TFs were identified related to fibre cell development at −1.5, −1, −0.5 and 0 DPA respectively. Each circle represents a gene, and triangle represents transcription factor. Node size is equivalent to the number of predicted connections. Node colour represents the weight abundance of predicted connections. Lines indicate edge weight (>0.01) for each pair of genes. Edge width represents the strength of the predicted connection.

Filtering the network down by TFs, four new TF regulatory networks were obtained (Figure 6). The core network components in latter three stages (−1, −0.5 and 0 DPA) were *MYB25‐like*, *HD1* and *HOX3* respectively. These three genes are superstars in fibre cell development (Cao *et al*., 2020; Shan *et al*., 2014; Walford *et al*., 2011, 2012; Wan *et al*., 2016), but the detailed temporal pattern of how they work in individual fibre cell has not yet been answered. After comparative analysis, *MYB25‐like_At* (Ghir_A12G017450) and *MYB25‐like_Dt* (Ghir_D12G017660) were only identified at −1 and −0.5 DPA network (Figure 6), as both of them have been reported associated with lint fibre development (Zhu *et al*., 2018). While *HD1* gene, who expressed predominantly in epidermal tissues during early fibre development (Walford *et al*., 2012), was identified in all these four stages. Meanwhile, the expression pattern of PROTODERMAL FACTOR 2 (*PDF2*) gene (Abe *et al*., 2003) was consistent with *HD1* (Figure 6). Another HD‐ZIP TF *HOX3*, its silencing greatly reduces fibre length whereas overexpression leads to longer fibre (Shan *et al*., 2014), was only identified at 0 DPA network (Figure 6). And also, a R2R3 MYB TF *MYB109*, who plays a role in fibre elongation (Pu *et al*., 2008), showed the same pattern as *HOX3*.

As core network components, MIXTA‐like MYB genes *MYB25* and *MML9* were only identified at −0.5 and 0 DPA networks (Figure 6). *MYB25* played a role in regulating specialized outgrowth of fibre cell (Machado *et al*., 2009). *MML9* was reported preferentially expressed during fibre initiation (Zhang *et al*., 2015). At −1.5 DPA, the core gene *WRKY44* (Ghir_A04G008530), also named *TTG2*, was reported in *Arabidopsis* involved in a regulatory module for regulation of seed coat mucilage synthesis (Xu *et al*., 2022).

### *MYB25‐like* determines fibre differentiation at −1 DPA and *HOX3* determines fibre tip‐biased diffuse growth at 0 DPA

**Figure 7:**
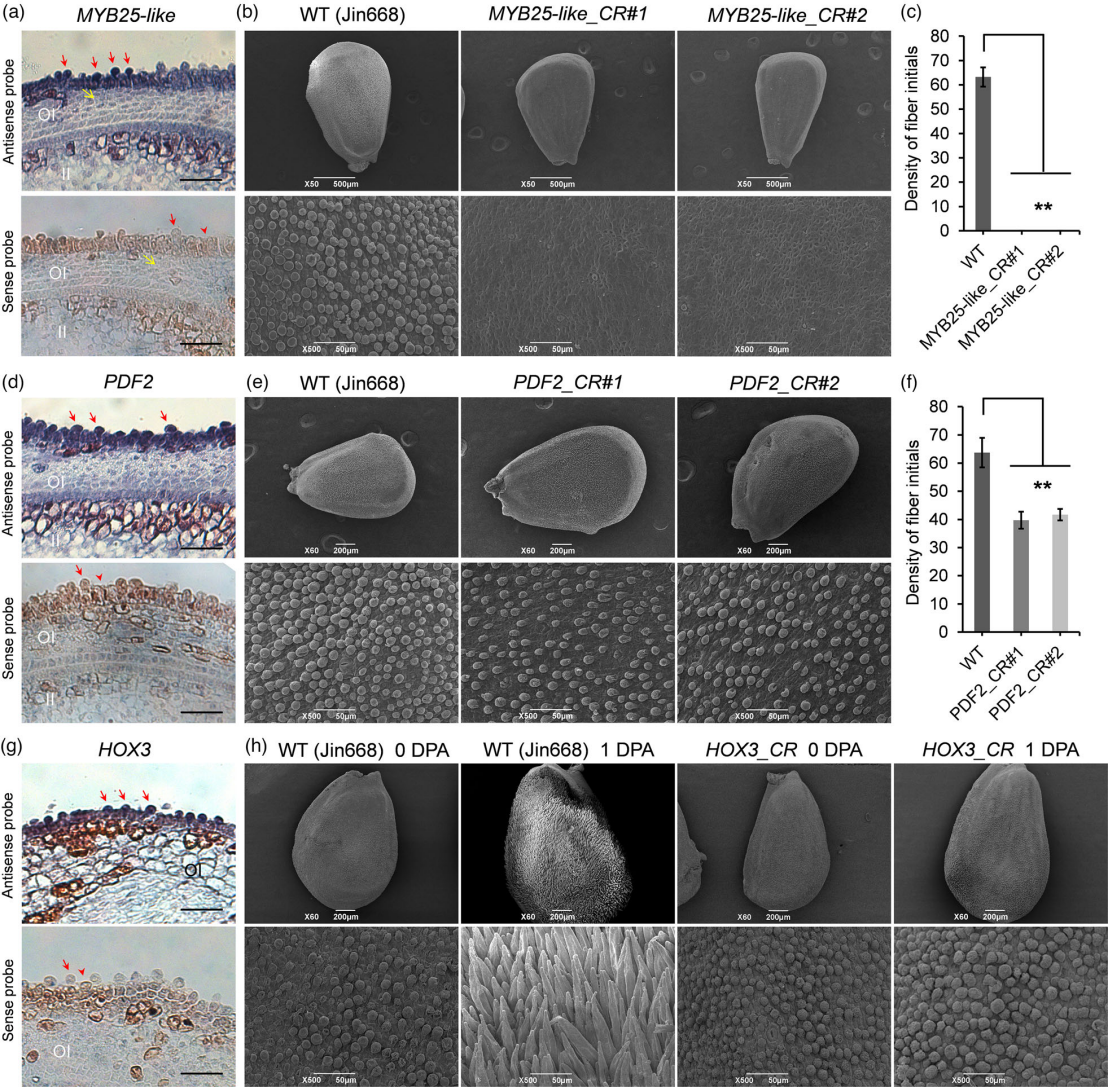
Function verification of cotton *MYB25‐like*, *PDF2* and *HOX3* genes. (a) *MYB25‐like* RNA *in situ* hybridization in LF_0d ovules. The sense probe was used as the negative control. OI, outer integument; II, inner integument. Red arrows indicate fibre cells, and red arrowheads indicate non‐fibre epidermal cells. Yellow arrows indicate outer pigment layer. Scale bars, 50 μm. (b) Fibre initials morphology between WT (Jin668, the transgenic receptor material) and two types of *MYB25‐like* CRISPR mutant (*MYB25‐like _CR*, both *MYB25‐like* homologues from At and Dt subgenomes knocked out) plants at 0 DPA. The upper panel shows the whole ovule, the lower panel is a magnified view of the ovule middle region. Scale bars, 500 μm (upper panel) and 50 μm (lower panel). (c) Statistics analysis of initial fibre densities (numbers per 0.01 mm^2^) on 0 DPA ovules between WT and *MYB25‐like_CR* plants. (d) RNA *in situ* hybridization of *PDF2* in LF_0d ovules with the sense probe as a negative control. OI, II, red arrows and red arrowheads have the same meaning as described in (a). Scale bars, 50 μm. (e) Fibre initials morphology in two types of *PDF2_CR* (both *PDF2* homologues from At and Dt subgenomes knocked out) lines and WT plants at 0 DPA. The lower panel is a magnified view of the middle region of whole ovule (upper panel). Scale bars, 200 μm (upper panel) and 50 μm (lower panel). (f) Statistics analysis of initial fibre densities (numbers per 0.01 mm^2^) on 0 DPA ovules between WT and *PDF2_CR* plants. All asterisks indicate significant differences when compared with the WT plants (*T*‐test, ** *P* < 0.01). (g) RNA *in situ* hybridization of *HOX3* in LF_0d ovules with the sense probe as a negative control. OI, II, red arrows and red arrowheads have the same meaning as described in (a). Scale bars, 50 μm. (h) Fibre initials morphology in WT and *HOX3_CR* (both *HOX3* homologues from At and Dt subgenomes knocked out) line at 0 DPA and 1 DPA respectively. The lower panel is a magnified view of the middle region of whole ovule (upper panel). Scale bars, 200 μm (upper) and 50 μm (lower).

*MYB25‐like* defunction was previously reported to be the determinant of cotton fibreless mutant (Walford *et al*., 2011; Wan *et al*., 2016). Histochemical localization of *MYB25‐like*‐GUS expression was localized in the epidermal layer of 0 DPA ovules (Walford *et al*., 2011). Here, compared to sense probe hybrid result, *MYB25‐like* expressed in ovule outer integument layer, including outer pigment layer and epidermis, and more highly in fibre cells (Figure 7a). Two independent lines with different editing types resulting both *MYB25‐like_At* and *MYB25‐like*_*Dt* knocked out were obtained (Figure S11a) and they showed totally fibreless phenotype (Figure S11b). Distinct from *GhMYB25‐like* RNA interference suppression lines, which still has few lint fibres attached on mature seeds (Walford *et al*., 2011), the seeds of our CRISPR mutant lines were totally glabrous. When observing 0 DPA ovules with SEM, no fibre initials was found in both *MYB25‐like_CR* lines as compared to WT (Jin668, the transgenic receptor material, Figure 7b,c).

Cotton *PDF2* was a member of HD‐ZIP class IV homeodomain protein family, which shares the highest homology with *AtPDF2* in *Arabidopsis*. *AtPDF2* and its paralogue *ATML1* are functionally interchangeable and act on *Arabidopsis* shoot epidermal cell differentiation (Abe *et al*., 2003; Rombola‐Caldentey *et al*., 2014). Cotton *PDF2* was validated highly expressed in ovule epidermis and fibre cells by *in situ* hybridization assays (Figure 7d). Two types of *PDF2* CRISPR mutant lines (*PDF2_CR*, both *PDF2* homologues from At and Dt subgenomes knocked out) were created (Figure S12a) and their fibre initials were significantly decreased on 0 DPA ovules as compared to WT (Figure 7e,f). However, the mature fibre phenotype and fibre quality of *PDF2_CR* lines were nearly the same as WT, except for a decrease in fuzz fibre density (Figure S12b‐c).

Cotton *HOX3* was highly expressed in 0 DPA fibre cells validated by *in situ* hybridization assays (Figure 7g). *HOX3* transcript level was sharply decreased in homozygous transgene co‐suppression lines, and resulting in retarded fibre elongation (Shan *et al*., 2014). Here, through CRISPR‐Cas9 based gene editing technology, the *HOX3* CRISPR mutant line (*HOX3_CR*, both *HOX3* homologues from At and Dt subgenomes knocked out) was obtained (Figure S13a). Compared to WT, the phenotype of *HOX3_CR* line was seemingly naked seeds (Figure S13b), different from the *HOX3* transgene co‐suppression lines which attached with very short fibres (Shan *et al*., 2014). When observed with SEM, normal fibre initials can be seen on 0 DPA ovules both in WT and *HOX3_CR* line (Figure 7h). At 1 DPA, fibre initials normally elongated on WT ovules but not on *HOX3_CR* line (Figure 7h).

Above all, the specific function of *MYB25‐like* and *HOX3* were more clearly clarified using CRISPR gene editing technology. *MYB25‐like* determines fibre differentiation, so defunctionalization of *MYB25‐like* produces no fibre initials; *HOX3* determines fibre tip‐biased diffuse growth, so cotton plants with *HOX3* defunctionalization can perform normal fibre differentiation and diffuse growth, but cannot transform into tip‐biased diffuse growth. Besides, *PDF2* play a role in fibre initiation, while it is not as critical as *MYB25‐like* and *HOX3*.

## Discussion

### The challenges of scRNA‐seq in non‐model crops

scRNA‐seq has flourished in plants (Ryu *et al*., 2021), but challenges remain, especially for non‐model crops. The biggest obstacle is definition for each cell type. In model plants *Arabidopsis* and poplars, a large number of known markers had been reported, which was enough to define almost all the cell types (Chen *et al*., 2021; Zhang *et al*., 2019). While in rice, corn and cotton (in this study), cell type definition still relies on RNA *in situ* hybridization (Liu *et al*., 2021b; Satterlee *et al*., 2020; Wang *et al*., 2021b; Xu *et al*., 2021), which is still a technologically dependent and time‐consuming challenge. Spatial transcriptome technology overcame this disadvantage and has been successfully applied in *Arabidopsis* leaves, which showed the *bona fide* single‐cell spatial transcriptome profiles (Xia *et al*., 2022). However, this technology still has many technical barriers in plant, for example, tissue optimization, due to the existence of cell wall.

The mutants are very effective to verify cell type definition. After scRNA‐seq on root tips of *rhd6* (lack root hair) and *gl2* (lack non‐hair cells) mutant, the cluster of root hair cells and non‐hair cells were respectively reduced compared to wild type (Ryu *et al*., 2019). The similar trend was observed in *Arabidopsis* root tips before and after heat stress treatment. For example, root hair cluster cell number was decreased in heat shock sample (Jean‐Baptiste *et al*., 2019). Here, we sequenced cotton Xu142 *fl* fibreless mutant ovules, and no fibre cell cluster was identified in Xu142 *fl* (Figure 4b). This not only proves the reliability of scRNA‐seq in cotton ovules but also confirms the accuracy of our cell type definition.

### Fibre cell initiation successively experiences differentiation, diffuse growth and tip‐biased diffuse growth

The study on fibre initiation started from last century (Stewart, 1975). The morning of anthesis (0 DPA) was always selected as a representative stage for fibre initiation (Haigler *et al*., 2012; Lee *et al*., 2007). And a lot of SEM observation and comparative transcriptome profiling was performed on 0 DPA ovules (Hu *et al*., 2018; Qin *et al*., 2019; Walford *et al*., 2012; Zhang *et al*., 2011). In this study, fibre initials can be observed on Xu142_LF line ovule epidermis at −0.5 DPA (Figure 1, Figure S1). Further, Xu142_LF fibre cell was identified differentiated at −1 DPA (Figure 5b). Therefore, with Xu142_LF line growing at Wuhan city, its −1 DPA samples should be collected for fibre differentiation study and −0.5 DPA samples for fibre diffuse growth study.

scRNA‐seq has been widely used in plants (Ryu *et al*., 2021; Seyfferth *et al*., 2021; Shaw *et al*., 2021), but reported studies were limited to one specific developmental stage, such as primary root tips of 5 days after germination in *Arabidopsis* (Ryu *et al*., 2019), root tips of 5 days rice seedlings (Zhang *et al*., 2021a), leaf blades of 7 days seedlings in peanut (Liu *et al*., 2021a), 5–10 mm developing ears of corn (Xu *et al*., 2021) and stem below the third internode of 4‐month‐old poplar (Chen *et al*., 2021). Here, our sampling strategy included four developmental stages during fibre initiation. In single sample, cell heterogeneity can be identified (Figure 2). In multiple samples, the elaborate developmental dynamics of fibre cell differentiation, diffuse growth and tip‐biased diffuse growth could be recognized (Figure 5).

**Figure 8:**
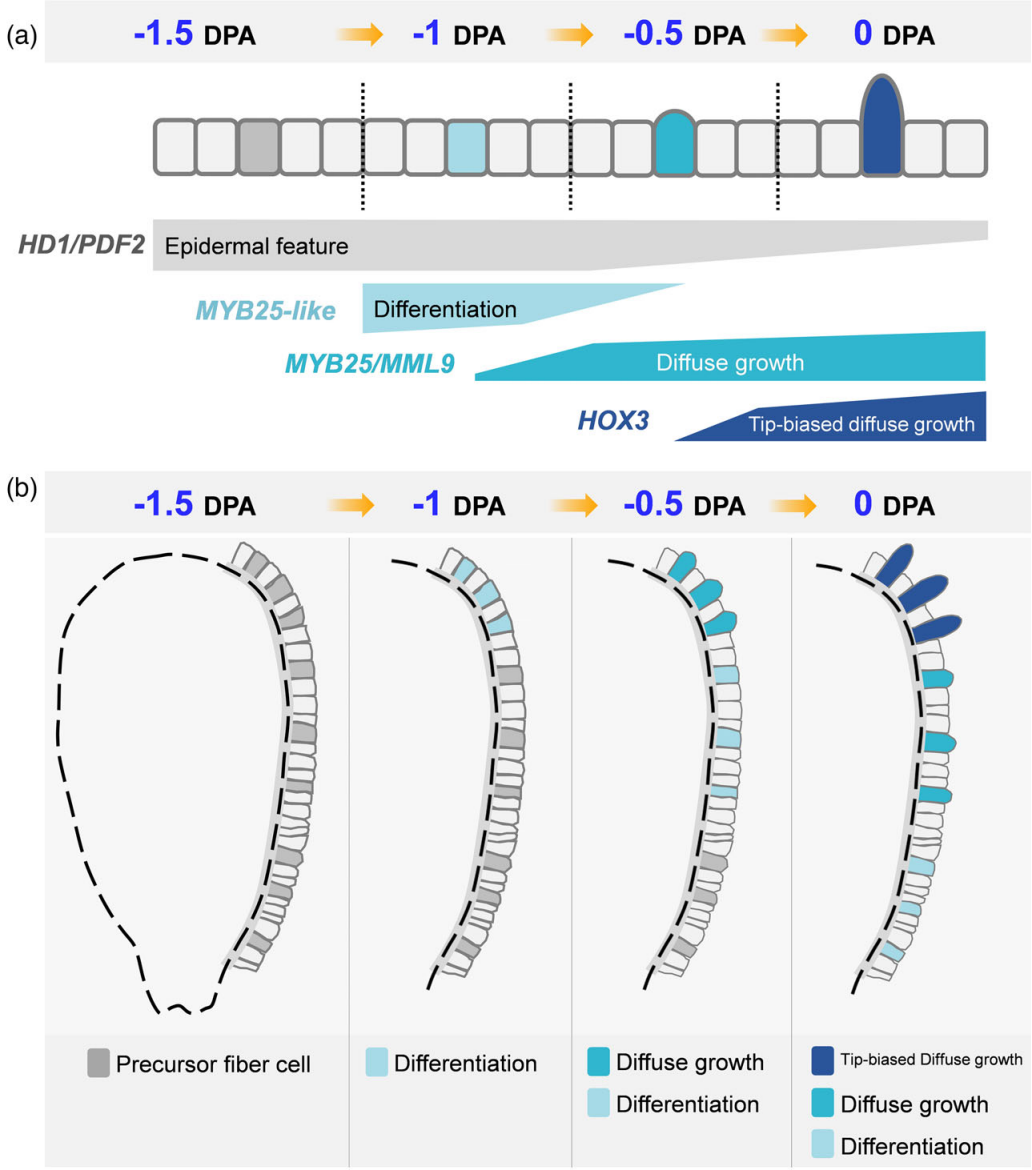
The proposed model for fibre initiation focusing on a single cell. (a) On cotton ovule epidermis, a precursor fibre cell successively experienced the process of differentiation, diffuse growth and tip‐biased diffuse growth during its initiation (above), as represented by four stages (−1.5, −1, −0.5 and 0 DPA). Different hub transcription factors that regulating each process are shown (below). (b) On the whole ovule, lint fibre differentiation firstly occurred on chalazal end at −1 DPA (marked by light sky blue). The differentiation progress gradually towards micropylar end. At −0.5 DPA, fibre initials could be seen at chalazal end (marked by cyan) due to its diffuse growth. At the same time, fibre differentiation has occurred in the middle of the ovule (light sky blue). At 0 DPA, the previously protruded fibre initials transformed into tip‐biased diffuse growth (dark blue), and the previously differentiated fibres began to protrude (cyan) due to diffuse growth. At the same time, fibre differentiation occurred at ovule micropylar end (light sky blue). To sum up, there were no fibre cell differentiated at −1.5 DPA; fibre differentiation occurred on chalazal end at −1 DPA; both fibre diffuse growth and differentiation occurred at −0.5 DPA; at 0 DPA, fibre tip‐biased diffuse growth, diffuse growth and differentiation occurred simultaneously.

Integrating fibre cell developmental trajectory, TF regulatory networks, and core network components functional validation, a model was proposed for fibre initiation focusing on a single cell (Figure 8a). A fibre cell successively experiences the process of differentiation, diffuse growth and tip‐biased diffuse growth, with different key regulators involved in each process. *HD1* and *PDF2* expressed at all four stages (Figure 6 and Figure 8a). They play roles in ovule epidermis before fibre differentiation, and also in fibre cell after then. *MYB25‐like* was identified preferentially expressed at −1 DPA (Figure 5e and Figure 6), promoting fibre cell differentiation. After fibre cell differentiated, *MYB25* and *MML9* start to express and gradually increased (Figure S7f,g), accompanying with fibre cell diffuse growth (Figure 8a). *MYB25* was likely regulated by MYB25‐like (Walford *et al*., 2011) then play a role in promoting fibre diffuse growth. At 0 DPA, a new core network component *HOX3* appeared (Figure 6) and promoted fibre tip‐biased diffuse growth (Figure 8a).

On whole ovule, fibre initiation firstly occurred on chalazal end and progressed gradually towards micropylar end (Figures 7 and 8b), according to earlier morphological observations (Stewart, 1975). Once fibre cell differentiated at −1 DPA, the continuous development leads to multiple processes occurring simultaneously on ovule epidermis (Figure 8b). As shown in re‐clustering result (Figure S7h), −1 DPA cells located only at the start of trajectory. At −0.5 DPA, cell distribution expanded (Figure S7h), means these cells including both early fibre cell and diffuse growing cells (Figure 8b). The 0 DPA cells were located at all the five sub‐clusters (Figure S7h), including cells in differentiation, diffuse growth and tip‐biased diffuse growth (Figure 8b). With scRNA‐seq, gene expression pattern in cells at different developmental stages can be more finely characterized, which is an important reason why this study can subdivide fibre initiation into three processes and identify the regulatory network of each process.

### *MYB25‐like* and *HOX3* play most important roles as commanders in fibre differentiation and fibre tip‐biased diffuse growth

Through cotton transgenic verification, *PDF2* gene mutation only decreased fibre initials at 0 DPA (Figure 7e). In *Arabidopsis*, single mutant of *AtPDF2* or its paralogue *ATML1* display normal shoot development, while double mutant results in severe defects in shoot epidermal cell differentiation (Abe *et al*., 2003; Rombola‐Caldentey *et al*., 2014). In cotton, *PDF2* homologues *GhHOX1* (Wang *et al*., 2004) and *GhHD1* (Walford *et al*., 2012) had been cloned and verified promoting fibre development. *PDF2* might also functionally interchangeable to *GhHOX1* or *GhHD1*, or both of them. So, double mutants on *PDF2* and its paralogue might display obvious phenotype in cotton.

There is no report to clarify the spatiotemporal pattern of *MYB25‐like* gene at single cell resolution. Here, expression pattern on fibre developmental trajectory showed that *MYB25‐like* was preferentially expressed in early fibre cells (Figure 5e). With qRT‐PCR measurements, *MYB25‐like* showed higher expression in −1 to 3 DPA ovules (Walford *et al*., 2011), in can be inferred that the expression of *MYB25‐like* mainly contributed by the newly differentiated fibre cells. On one hand, *MYB25‐like* gene starts to be highly expressed at −1 DPA (Walford *et al*., 2011), which is consistent with our result (Figure 6). On the other hand, its high expression lasts until 3 DPA, a stage when fuzz fibre begins to differentiate, which indicated that *MYB25‐like* is also participated in fuzz initiation.

Hormone response and sugar signal genes were reported involved in fibre initiation (Huang *et al*., 2021; Tian and Zhang, 2021; Wang *et al*., 2021a). Interestingly, *MYB25‐like* was down‐regulated in fibre‐deficient mutants of hormone‐ or sugar‐signalling genes. For example, *MYB25‐like* activity was suppressed in *GhJAZ2* overexpressing lines (Hu *et al*., 2016); the transcripts of *MYB25‐like* was dramatically reduced in *GhVIN1*‐RNAi lines (Wang *et al*., 2014) and down‐regulated in *GhPIN1a*‐RNAi ovules (Zhang *et al*., 2017); *MYB25‐like* was severely down‐regulated in ovules cultured with excess ZT (*trans*‐zeatin, a kind of cytokinin) (Zeng *et al*., 2019). These suggested that hormone and sugar signalling may act on the upstream of *MYB25‐like* and through this gene to exert their effects on fibre development. At least among currently known fibreless mutants (such as Xu142 *fl*, *GhVIN1*‐RNAi lines and *GhPIN1a*‐RNAi lines) (Walford *et al*., 2011; Wang *et al*., 2014; Zhang *et al*., 2017), *MYB25‐like* gene was the direct commander controlling fibre cell differentiation.

*HOX3* gene highly expressed at 0 DPA (Figure 6) and mainly in fibre cells (Figures 2c and 7g). Fibre cell can normally differentiate and diffuse grow but cannot transform into tip‐biased diffuse growth in *HOX3_CR* line (Figure 7h). Therefore, *HOX3* seems to act as a commander controlling fibre cell development transformation. As shown in Figure 8a, two commanders (*MYB25‐like* and *HOX3*) positively regulating early fibre development are functioning like a ‘relay race’ model. The RNA‐seq, chromatin immunoprecipitation sequencing (ChIP‐seq) with these cotton mutants and identification of TF regulatory multiple complexes have been ongoing in our lab, the molecular mechanism hidden below ‘relay race’ model will be revealed in the near future.

### Core regulators controlling the differentiation and tip‐biased diffuse growth of *Arabidopsis* trichomes and cotton fibres may be conserved

In *Arabidopsis*, mutant of two genes, *GL1* (R2R3‐type MYB transcription factor GLABRA1) and *GL2* (homeobox transcription factor GLABRA2), produced no leaf trichomes (Hulskamp *et al*., 1994; Szymanski *et al*., 1998), but they were functioning in different ways. For *gl2* leaves some large cells bigger than normal epidermal cells appeared and their nuclei were also in the same size as wild‐type trichomes, which means trichome cells can differentiate and enlarge, but no local outgrowth (Hulskamp *et al*., 1994). However, all the epidermal cells of *gl1* leaves were uniform in size and shape, and no specialized cells was found, which suggested that *GL1* is required for trichome cell differentiation (Hulskamp *et al*., 1994). In this aspect, *MYB25‐like* gene functions similarly to *GL1*, and *HOX3* to *GL2*. Because they were in same gene family respectively, but also the similar ways they acting on fibre cell or trichomes. In addition, other signals affecting trichome development such as phytohormones (Qi *et al*., 2011; Qi *et al*., 2014; Zhou *et al*., 2013), miRNAs (Xue *et al*., 2014; Yu *et al*., 2010), R3 MYBs (Gan *et al*., 2011; Vadde *et al*., 2019; Wang *et al*., 2007; Zhao *et al*., 2008) or histone demethylase (Hung *et al*., 2020), were always acting on the upstream of *GL1*. This also showed a similar pattern as cotton *MYB25‐like*. Therefore, the core regulators controlling differentiation and tip‐biased diffuse growth of *Arabidopsis* leaf trichomes and cotton fibres appear to be conserved. This also provides some inspiration for exploring other types of epidermal tissue, such as tomato trichomes, kapok fibre, tea trichomes, and okra seed coat mucilage.

## Conclusion

In summary, with the help of scRNA‐seq technology and reasonable multi‐stage sampling strategy, fibre cell differentiation, diffuse growth and tip‐biased diffuse growth process during fibre initiation were more finely delineated. Through gene regulatory network analysis and CRISPR‐Cas9‐based gene function verification, the *MYB25‐like* gene was newly defined as a commander acting at −1 DPA on fibre cell differentiation. In addition, *HOX3* was proved to be another commander controlling fibre development transformation into tip‐biased diffuse growth. It is the first report by applying scRNA‐seq technology in cotton fibre cell, and our result provides a more refined and detailed stage definition of fibre initiation. The valuable resource provided here will help to further explore the mechanism of fibre development, plant trichomes differentiation and single cell fate determination.

## Materials and methods

### Plant growth and sample collection

The cotton Lint‐Fuzz (Xu142_LF) line, derived from recombinant inbred lines of Xu142 × Xu142 *fl* (Hu *et al*., 2018), was planted in the experimental field at Huazhong Agriculture University, Wuhan, China. Cotton plants were grown under conventional field management. Samples were collected when cotton plants began flowering. −1 DPA flower buds, 0 DPA flowers were collected in the morning (8 : 00 a.m.), and −1.5 DPA and −0.5 DPA buds were collected in the evening (8 : 00 p.m.).

### Scanning electron microscopy (SEM) and transmission electron microscopy (TEM)

Cotton bolls of Xu142_LF were collected at −1.5 DPA and every 4 h from −1 DPA to 0 DPA (i.e. 8 : 00, 12 : 00, 16 : 00, 20 : 00, 24 : 00 at −1 DPA and 4 : 00, 8 : 00 at 0 DPA). Ovules were fixed for further SEM observation. The detailed processes were performed as reported (Hu *et al*., 2018).

Ovules at −1.5, −1, −0.5 and 0 DPA were collected and fixed with 2.5% glutaraldehyde, followed by fixing with 1% OsO_4_, acetone gradient dehydration, resin infiltration and embedment. Sections of 90 nm were cut, counterstained, then visualized using a TEM (New Bio‐TEM H‐7500, HITACHI, Japan), according to the methods reported (Liu *et al*., 2015; Min *et al*., 2013).

### Tissue digestion and scRNA‐seq library preparation

Ovules collected from Xu142_LF at −1.5, −1, −0.5 and 0 DPA, and from 0 DPA of Xu142 and Xu142 *fl* were respectively placed into a 30‐mm‐diameter Petri dish containing 3 mL enzyme solution (1.5% [w/v] cellulose [‘ONOZUKA’ R‐10, Yakult], 1% [w/v] hemicellulose [Sigma‐Aldrich], 0.75% [w/v] Macerozyme [R‐10, Solarbio], 0.4 M Mannitol, 20 mm MES [pH 5.7], 20 mm KCl, 10 mm CaCl_2_, 0.1% [w/v] bovine serum albumin (BSA)). Four cotton balls were harvested and all ovules in these balls were collected together as one sample. Petri dishes were placed in a vacuum drying oven and kept at 0.1 atmospheric pressure for 5 min to promote the removal of gases in the ovule to facilitate the full submersion of the ovule in the enzyme solution. The dish was rotated at 60 rpm for 4 h at 25°C.

The enzyme solution was filtered with a 40 μm cell strainer, 1× PBS solution containing 0.04% BSA was added to the filtrate, gently inverted and mixed, centrifuged at 100 rcf for 2 min, and the supernatant was carefully discarded. Filtering, rinsing, and centrifuging steps was repeated twice to obtain a single cell suspension. The cell suspension was kept on ice to prevent cell death. Protoplast concentration was determined using a haemocytometer, and the ideal concentration is 700–1200 cells/μl. Protoplasts were stained with 0.4% trypan blue solution for detecting viability.

A commercially available droplet‐based system from 10× Genomics Inc. (Zheng *et al*., 2017) was used to isolate protoplasts. The protoplast suspension was loaded into Chromium microfluidic chips with 3′ v2 chemistry. RNA from the barcoded cells was subsequently reverse‐transcribed, then sequencing libraries were constructed and sequenced on NovaSeq (Illumina) platform using Hiseq PE150 strategy.

### Processing of scRNA‐seq data

The detailed calculation methods and parameter settings involved in this process are described in Appendix S1. In brief, the raw data in FASTQ format were first processed to obtain clean reads. *Gossypium hirsutum* TM‐1 genome (Wang *et al*., 2019) was used as reference genome. Only reads that were uniquely mapped were used for UMI counting. The Seurat package (v. 4.0.1) implemented in R (v. 4.0.0) was used for gene‐cell matrices analysis, including doublets, no‐load cells and dead cells filtration. Principal Components Analysis (PCA), Uniform Manifold Approximation and Projection (UMAP) and *t*‐Stochastic Neighbour Embedding (*t*‐SNE) analyses were performed for visualizing data in 2‐d space. Cluster‐enriched genes were identified with Seurat function ‘FindAllMarkers’. The subset of related clusters was extracted and processed for pseudo‐time analysis. The weighted gene co‐expression network analysis (WGCNA) was performed in R software (v. 4.0.0) following the official process. The gene regulatory network displaying, personalized Gene Ontology (GO) and pathway enrichment analysis were performed using OmicShare tools, a free online platform for data analysis (www.omicshare.com/tools).

### Analysis of RNA‐seq data

Bulk RNA‐seq data of Xu142_LF 0 DPA ovule outer integument from our previous study (Hu *et al*., 2018) was used here. Clean reads were newly mapped to the updated TM‐1 reference genome (Wang *et al*., 2019) using Hisat2 (v2.1.0) software (Kim *et al*., 2015). The mapping reads were sorted to filter those reads representing PCR duplicates. Sequencing reads with mapping quality of <25 were filtered using SAMTOOLS (v.0.1.19; Li *et al*., 2009), the remaining were used to calculate gene expression levels using STRINGTIE software (v.1.3.4) with default settings (Pertea *et al*., 2015).

### RNA *in situ* hybridization

*In situ* hybridization was carried out as described in cotton research previously (Zhang *et al*., 2017). Briefly, 0 DPA ovaries of Xu142_LF were collected and embedded in paraffin. 10 μm paraffin sections were de‐paraffinized, rehydrated and incubated overnight with the Dig‐labelled RNA probe (Roche). Sections were then incubated with alkaline phosphatase‐conjugated anti‐digoxigenin (anti‐Dig‐AP, Roche) and the signal was detected by nitro‐blue tetrazolium/5‐bromo‐4‐chloro‐3‐inodyl‐phosphate (NBT/BCIP) colour substrate solution (Roche). Sections incubated with sense RNA probe were used as negative control. Images were captured using fully motorized upright microscope (Leica DM6B) in bright‐field mode. Primers are listed in Table S12.

### Vector construction and plant transformation

CRISPR technology was employed to create the *GhMYB25‐like‐CR* (Ghir_A12G017450*/*Ghir_D12G017660), *GhHOX3‐CR* (Ghir_A12G028530/Ghir_D12G028680) and *GhPDF2‐CR* (Ghir_A10G001030/Ghir_D10G001810) mutants in cotton. For targeting *MYB25‐like* genes, two sgRNAs (CTCCATGTAGCGACAAGGTG; CGCCCTTCTTGGAAACAGGT) were designed for targeting MYB25‐like. The primers listed in the Table S12 were used to amplify tRNA and gRNA from the template pGTR vector. Two different fragments containing tRNA‐sgRNA1 and gRNA‐tRNA‐sgRNA2 were integrated together by an overlapping extension PCR. Finally, the PCR products were purified and inserted the Bsa I‐digested pRGEB32‐GhU6.9 vector using ClonExpress II One Step Cloning Kit (Vazyme) (Wang *et al*., 2018). For targeting *GhHOX3* (ACCGGTACAACCTGTTCATA) and *GhPDF2* (CAACTGTGTCTCCTTACTTA) genes, single sgRNA was used in the *GhHOX3‐CR* and *GhPDF2‐CR* vector. The positive vectors were transformed into *Agrobacterium tumefaciens* strain EHA105 for cotton transformation. JIN668 was the transgenic receptor (Li *et al*., 2019).

### On‐target analysis of gene‐edited plants

T_0_ positive transgenic plants were screened by PCR analysis using Cas9 forward and reverse primers (Table S12). For identification of mutated alleles in T_1_ transgenic lines, high‐throughput (Hi‐Tom) sequencing was adopted (Liu *et al*., 2019). First, the targeted regions were amplified by PCR using site‐specific primers. Second, barcode primers were used to add barcodes to the first‐round PCR products. After the second‐round PCR amplification, the products of all samples were mixed in equal amounts and purified to perform next‐generation sequencing (NGS). Finally, NGS data was analysed by Hi‐Tom platform (http://hi‐tom.net/hi‐tom/).

## Conflicts of interest

The authors declared that they have no conflict of interest.

## Author contributions

L.T. and X.Z. conceived the project and designed the experiments. Y.Q. performed protoplast isolating, M.S. performed the transgenic experiments. Y.Q., M.S. and L.S. performed *in situ* hybridization experiment. Y.Q., M.S. and J.Y. performed bioinformatics analysis. Z.Y., W.L., J.X., M.X., L.S., Y.L. and G.Z. contributed to the vector construction. Y.Q., Z.L. and Z.X. built a website for data sharing. Y.Q., M.S. and L.T. wrote the manuscript, X.Y., M.W., K.L. and X.Z. revised the manuscript with feedback from all other authors.

## Supporting information


**Figure S1** Dynamic changes on Xu142_LF ovule epidermis during fibre initiation.
**Figure S2** Tissue digestion and protoplast isolation.
**Figure S3** Comparison between single‐cell RNA‐seq and bulk RNA‐seq of Xu142_LF.
**Figure S4** Expression and identification of cluster enriched genes.
**Figure S5** RNA *in situ* hybridization of cell‐type representative genes.
**Figure S6** Single‐cell RNA‐seq and clusters identification on Xu142 and Xu142 *fl*.
**Figure S7** Fibre cell cluster identification on the LF combined sample.
**Figure S8** Clustering and annotation of four LF samples.
**Figure S9** Hierarchical cluster tree showing co‐expression modules identified by WGCNA.
**Figure S10** Networks related to fibre cell initiation.
**Figure S11** Characterization of cotton CRISPR editing lines on *MYB25‐like* genes.
**Figure S12** Characterization of cotton CRISPR editing lines on *PDF2* genes.
**Figure S13** Characterization of cotton CRISPR editing lines on *HOX3* genes.

**Table S1** Summary of single‐cell RNA sequencing results for each sample.

**Table S2** Summary of protoplasting induced differentially expressed genes.

**Table S3** List of enriched genes for each cluster.

**Table S4** Cell number of each cluster identified from Xu142‐vs‐Xu142 *fl*.

**Table S5** KEGG analysis of LF_0d fibre cell enriched genes.

**Table S6** Cell barcode mapping between single sample and combined sample.

**Table S7** Summary of 167 branch‐dependent genes.

**Table S8** GO enrichment analysis of 167 branch‐dependent genes.

**Table S9** Summary of cluster‐enriched genes.

**Table S10** WGCNA on four LF samples.

**Table S11** Weight coefficient of fibre development‐related genes.

**Table S12** The primers used in this study.

**Appendix S1** Details of bioinformatics analysis.

## Data Availability

The scRNA‐seq data have been deposited in the NCBI SRA database (https://www.ncbi.nlm.nih.gov/bioproject/) with BioProject number PRJNA600131.
