# Supplementary material for: Single‐cell RNA‐seq reveals fate determination control of an individual fibre cell initiation in cotton (Gossypium hirsutum)
Source: Plant Biotechnol J. 2022 Oct 2;20(12):2372–88. doi: 10.1111/pbi.13918 (PMC9674311; doi:10.1111/pbi.13918)
Supplement: Supplementary file 1 — Figure S1 Dynamic changes on Xu142_LF ovule epidermis during fibre initiation. Figure S2 Tissue digestion and protoplast isolation. Figure S3 Comparison between single‐cell RNA‐seq and bulk RNA‐seq of Xu142_LF. Figure S4 Expression and identification of cluster enriched genes. Figure S5 RNA in situ hybridization of cell‐type representative genes. Figure S6 Single‐cell RNA‐seq and clusters identification on Xu142 and Xu142 fl. Figure S7 Fibre cell cluster identification on the LF combined sample. Figure S8 Clustering and annotation of four LF samples. Figure S9 Hierarchical cluster tree showing co‐expression modules identified by WGCNA. Figure S10 Networks related to fibre cell initiation. Figure S11 Characterization of cotton CRISPR editing lines on MYB25‐like genes. Figure S12 Characterization of cotton CRISPR editing lines on PDF2 genes. Figure S13 Characterization of cotton CRISPR editing lines on HOX3 genes. [file PBI-20-2372-s011.pdf]

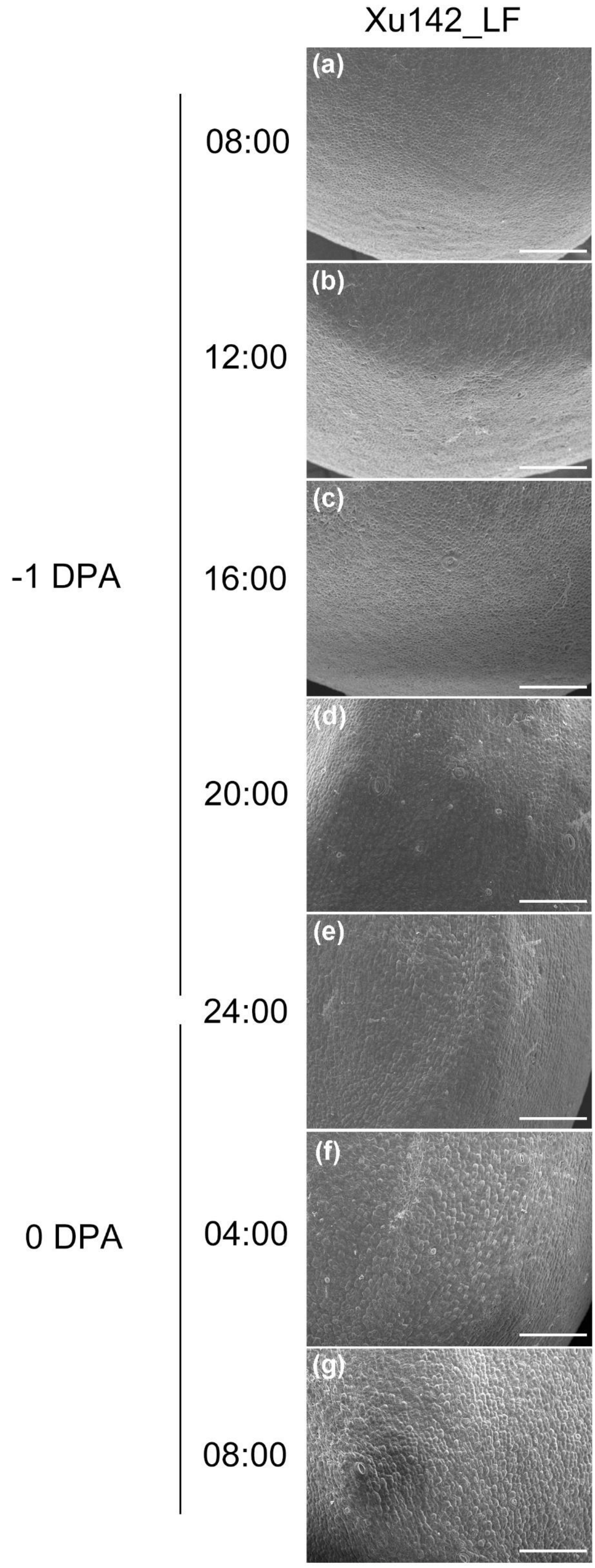

Fig.S1 Dynamic changes on Xu142_LF ovule epidermis during fiber initiation. (a-g) Ovule epidermis observed by Scanning Electron Microscopy (SEM) every four hours from -1 DPA (8:00 a.m.) to 0 DPA (8:00 a.m.). Scale bars, 100 μm.

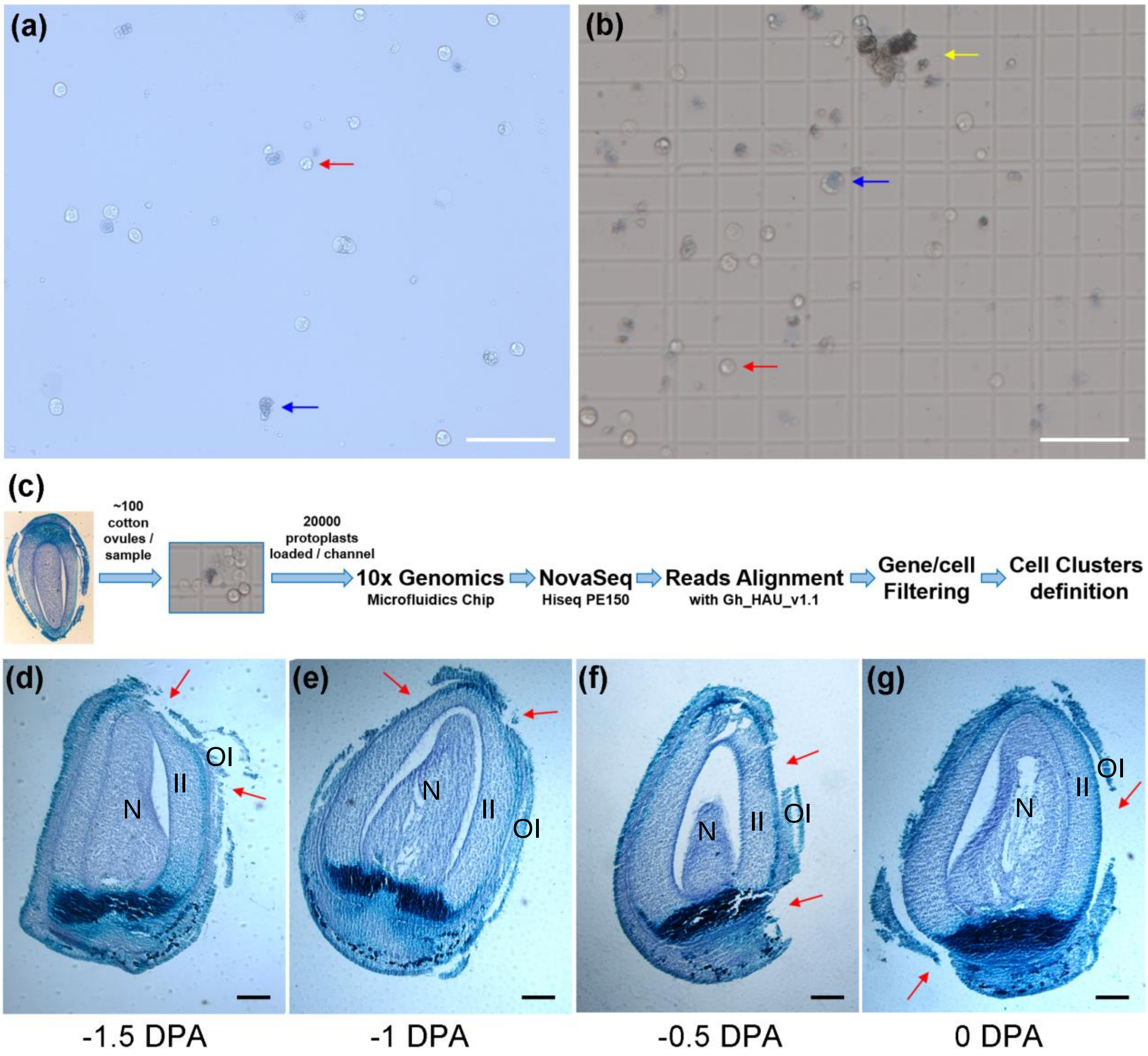

Fig.S2 Tissue digestion and protoplast isolation.

(a-b) Representative pictures after enzyme digestion showing alive cells (red arrow), dead cells (blue arrow) and some occasional undigested tissue pieces (yellow arrow). Scale bars, 100 μm. **(c)** Workflow used for scRNA-seq to obtain transcriptomes from individual cells. **(d-g)** Morphology observation of enzymatically hydrolyzed ovules (from -1.5 to 0 DPA) by longitudinal section and toluidine blue staining. Red arrows point to the part that's being enzymatically hydrolyzed. OI, outer integument; II, inner integument; N, nucellus. Scale bars, 200 μm.

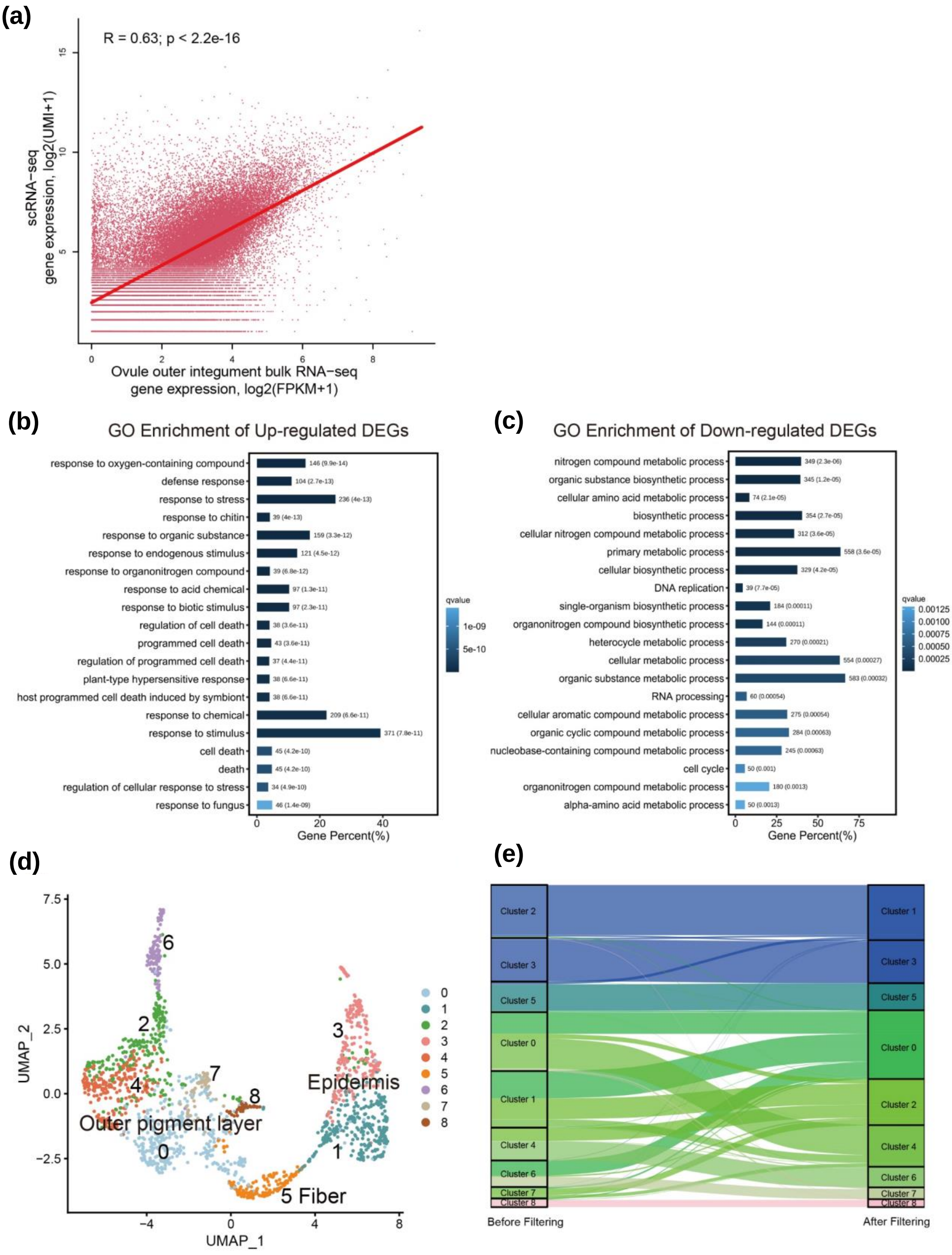

Fig.S3 Comparation between single-cell RNA-seq and bulk RNA-seq of Xu142_LF.

(a) Scatter plots showing the correlations between combined scRNA-seq and unprotoplasted ovule outer integument bulk RNA-seq. **(b-c)** GO enrichment analysis of up-regulated genes (**b**) and down-regulated genes **(c)** in response to protoplasting. **(d)** UMAP visualization of putative 9 clusters with cell-type annotation on LF_0d sample after removing differentially expressed genes induced by protoplasting process. **(e)** Sankey diagram showing the clustering cells before and after protoplasting induced genes filtered.

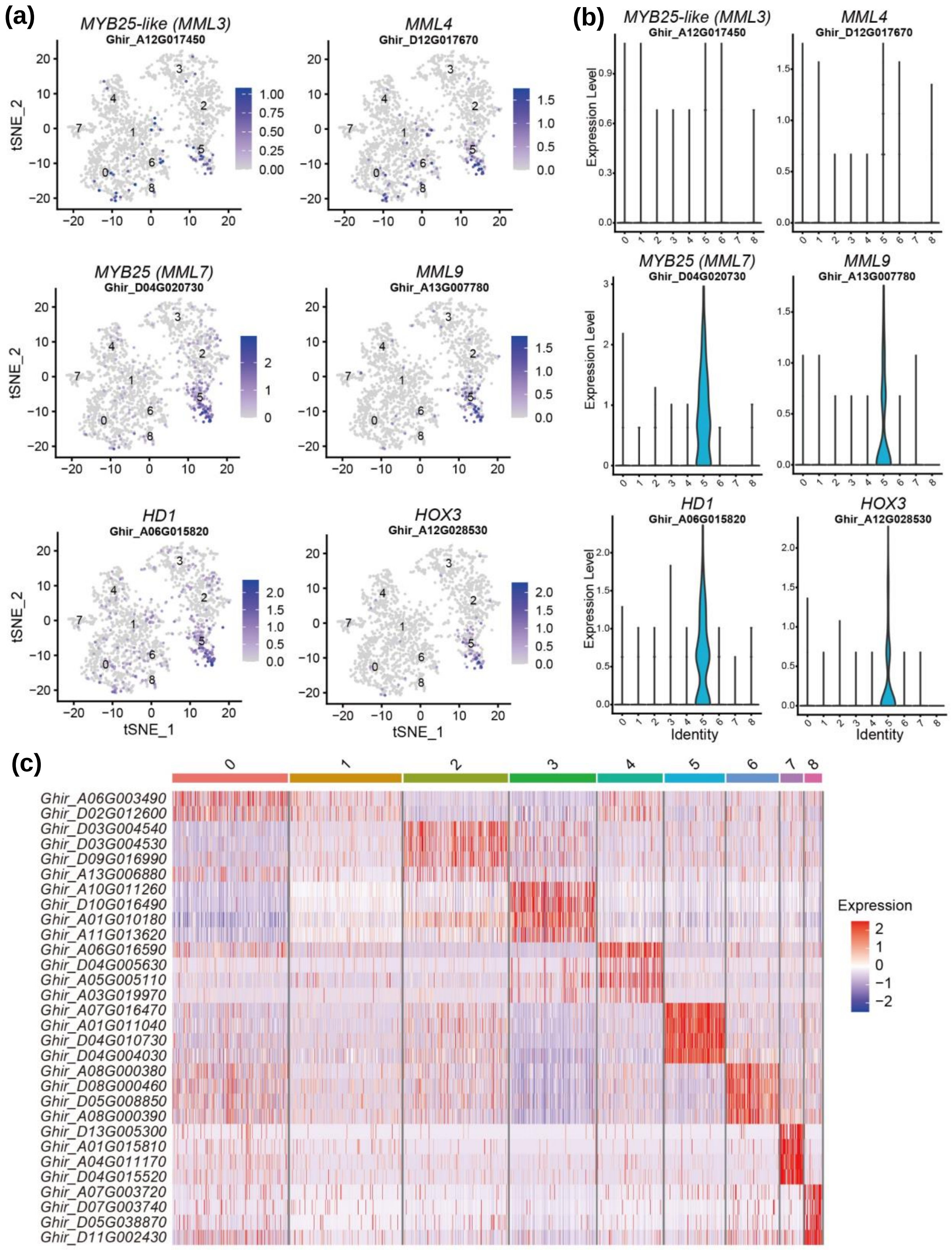

Fig.S4 Expression and identification of cluster enriched genes.

(a) *t*-SNE projection plots showing transcript accumulation for known fiber marker genes in individual cells. **(b)** Violin plot showing the expression pattern of known fiber markers in 9 cell clusters. **(c)** Heatmap showing the expression pattern of top 4 genes enriched in each cluster in LF_0d. Color bar indicate scaled expression level.

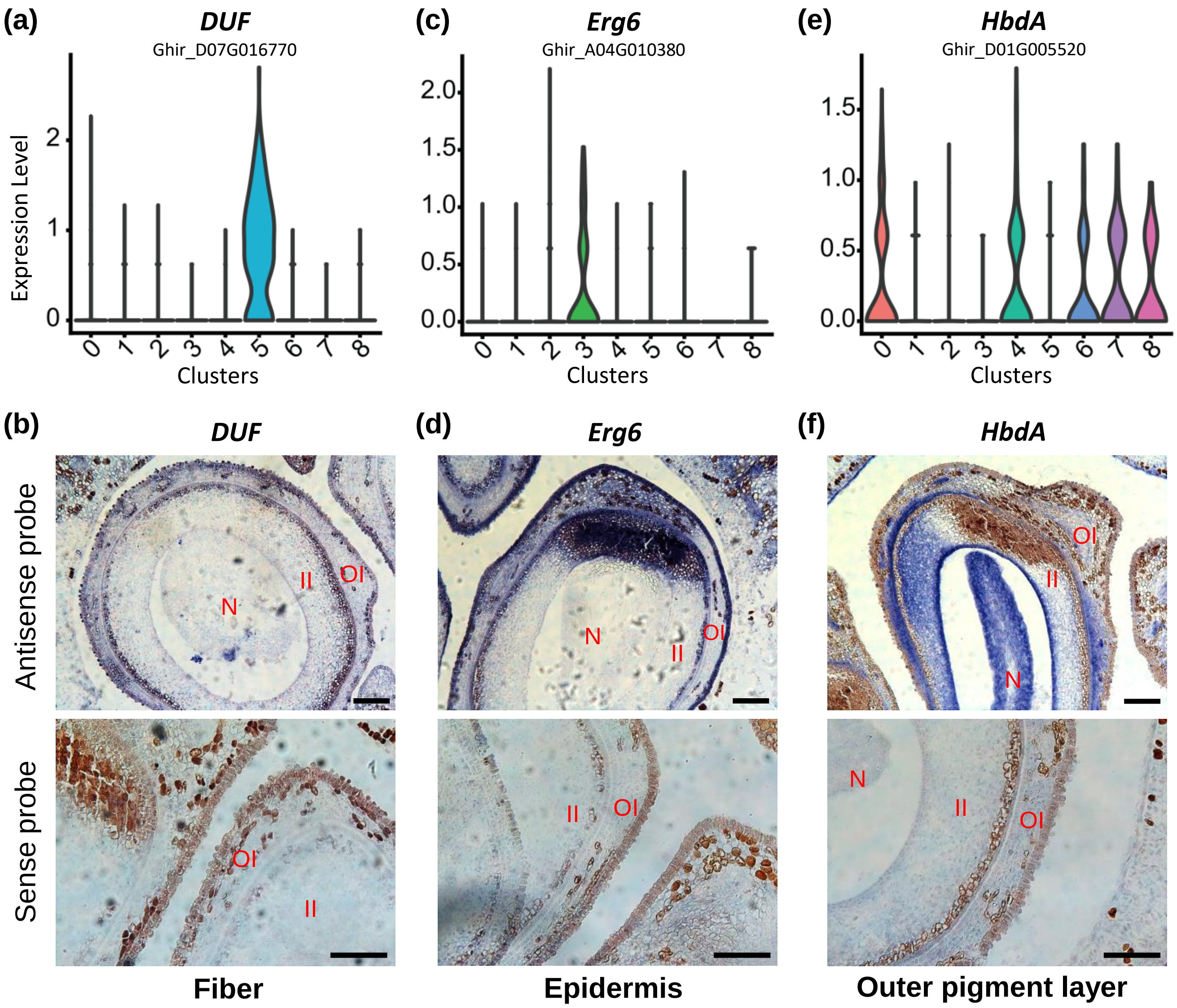

Fig.S5 RNA *in situ* hybridization of cell-type representative marker genes.
(a) Violin plot showing the expression pattern of a novel fiber marker gene *DUF* (*Ghir_D07G016770*) from cluster 5. **(b)** RNA *in situ* hybridization of fiber marker *DUF* with the sense probe as a negative control. **(c)** Expression of novel non-fiber epidermis marker gene *Erg6* (*Ghir_A04G010380*) in cluster 2 and 3 at violin plot. **(d)** RNA *in situ* hybridization of *Erg6* with the sense probe as a negative control. **(e)** Violin plot showing the expression pattern of a novel outer pigment layer marker gene *HbdA* (*Ghir_D01G005520*) in cluster 0, 4, 6, 7, 8. **(f)** RNA *in situ* hybridization of *HbdA* with the sense probe as a negative control. The enlarged views of these three hybridization signals were shown in Fig. 3. Sections (10 μm) from Xu142_LF ovules at 0 DPA were used for *in situ* hybridization. OI, outer integument; II, inner integument; N, nucellus. Scale bars, 100 μm.

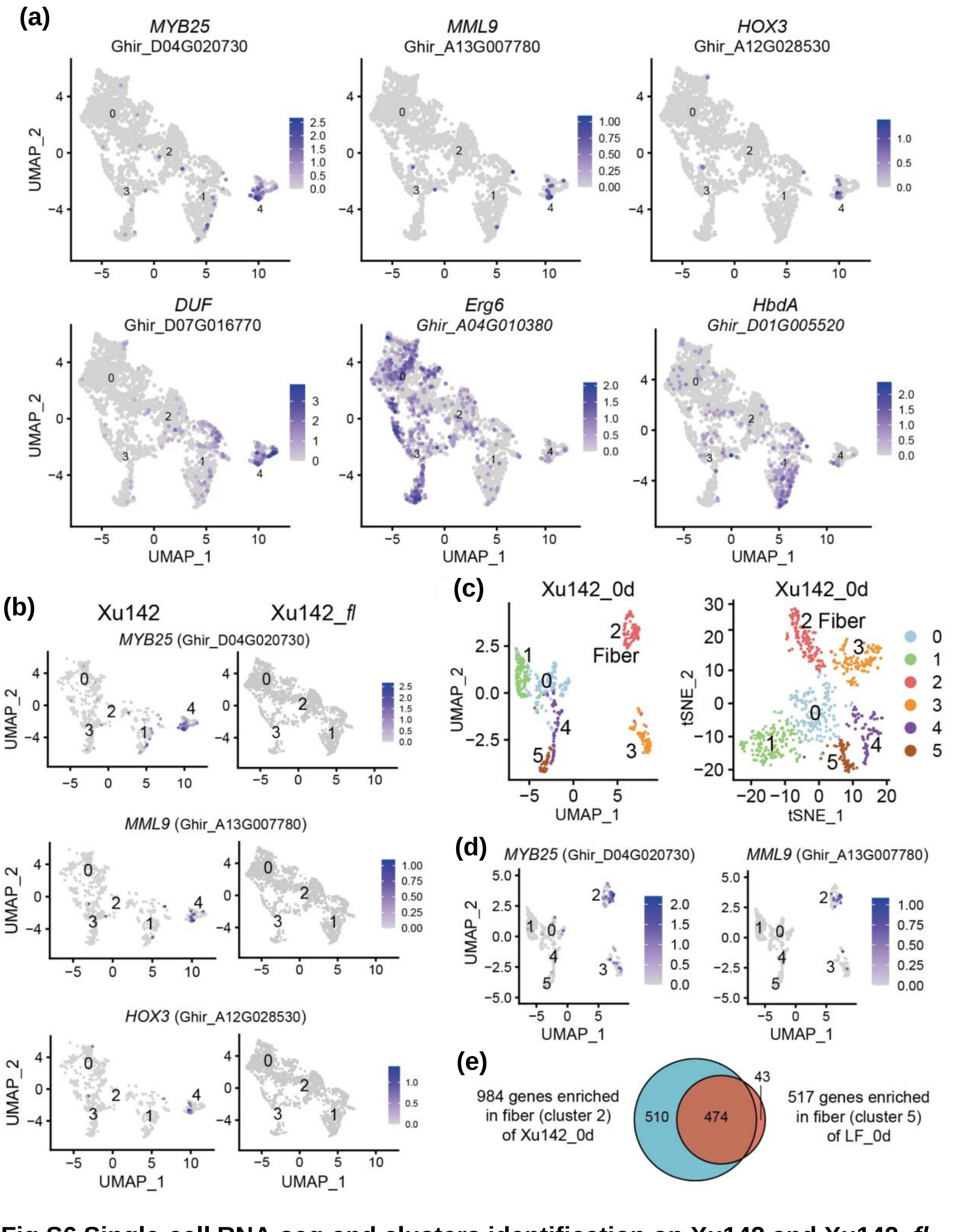

Fig.S6 Single-cell RNA-seq and clusters identification on Xu142 and Xu142_*fl*. **(a)** UMAP visualization of representative cell-type marker genes in Xu142-vs-Xu142_*fl*. *MYB25*, *MML9* and *HOX3* are known fiber markers. *DUF*, *Erg6* and *HbdA* were proved to be markers of fiber, epidermis and outer pigment layer, respectively (See Fig. S5). **(b)** The expression distribution of three known fiber markers displayed separately according to Xu142 and Xu142_*fl*. **(c)** UMAP and *t*-SNE projection plot showing 6 clusters from ovule outer integument of Xu142 0 DPA sample. **(d)** UMAP projection plots showing transcript accumulation for known fiber markers (*MYB25* and *MML9*) in individual cells. **(e)** Venn diagram showing the number of genes enriched in fiber cluster shared between LF_0d and Xu142_0d.

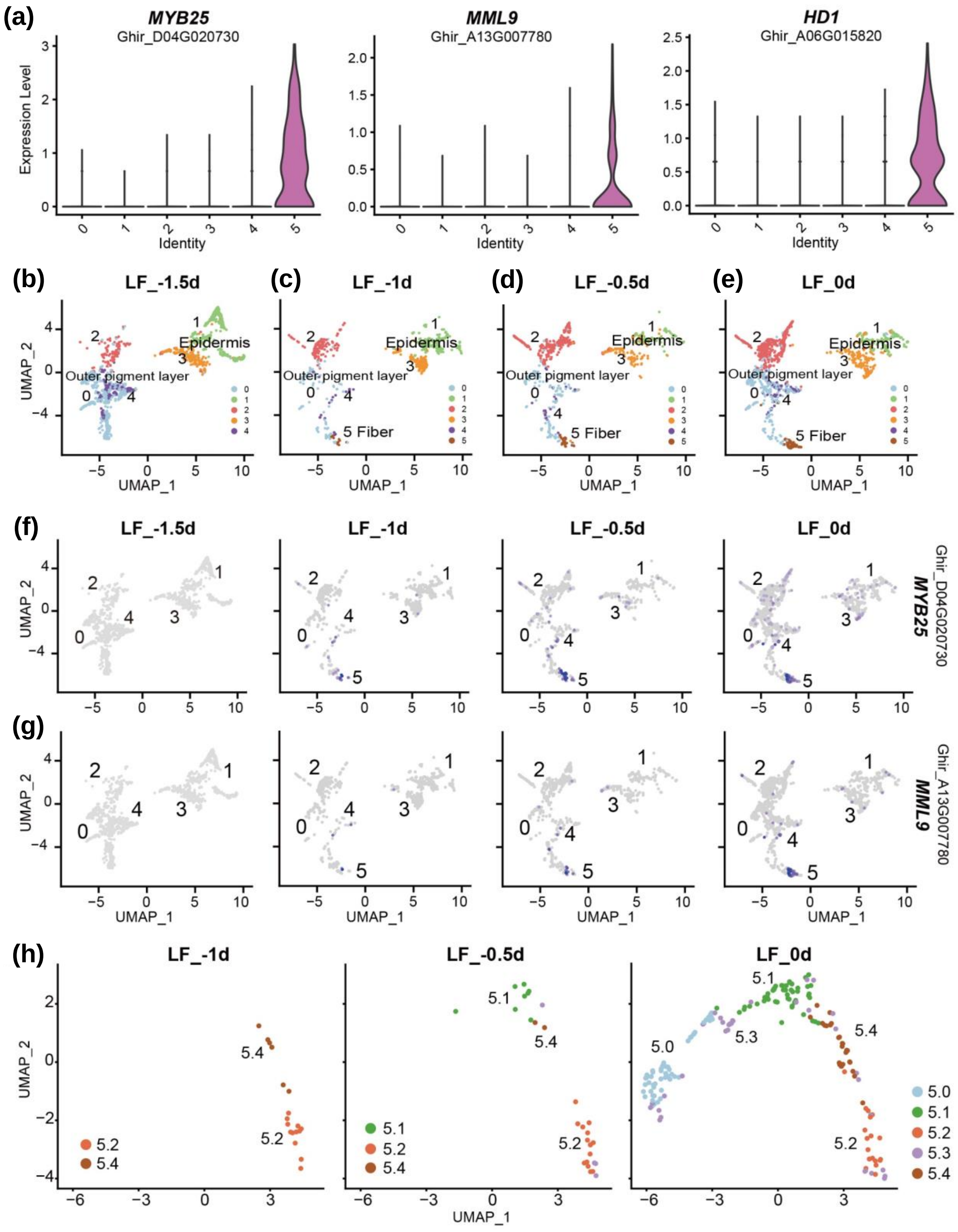

Fig.S7 Fiber cell cluster identification on the Xu142_LF combined sample.
(a) Violin plot showing the expression pattern of three fiber marker genes (*MYB25*, *MML9* and *HD1*) in 6 cell clusters. **(b-e)** UMAP visualization of putative clusters which shown separately at -1.5, -1, -0.5 and 0 DPA, respectively. **(f-g)** UMAP projection plots showing transcript accumulation for two fiber marker genes (*MYB25* and *MML9*) in individual cells separated according to 4 stages. **(h)** UMAP visualization of putative clusters of selected fiber cells which shown separately at -1, -0.5 and 0 DPA, respectively.

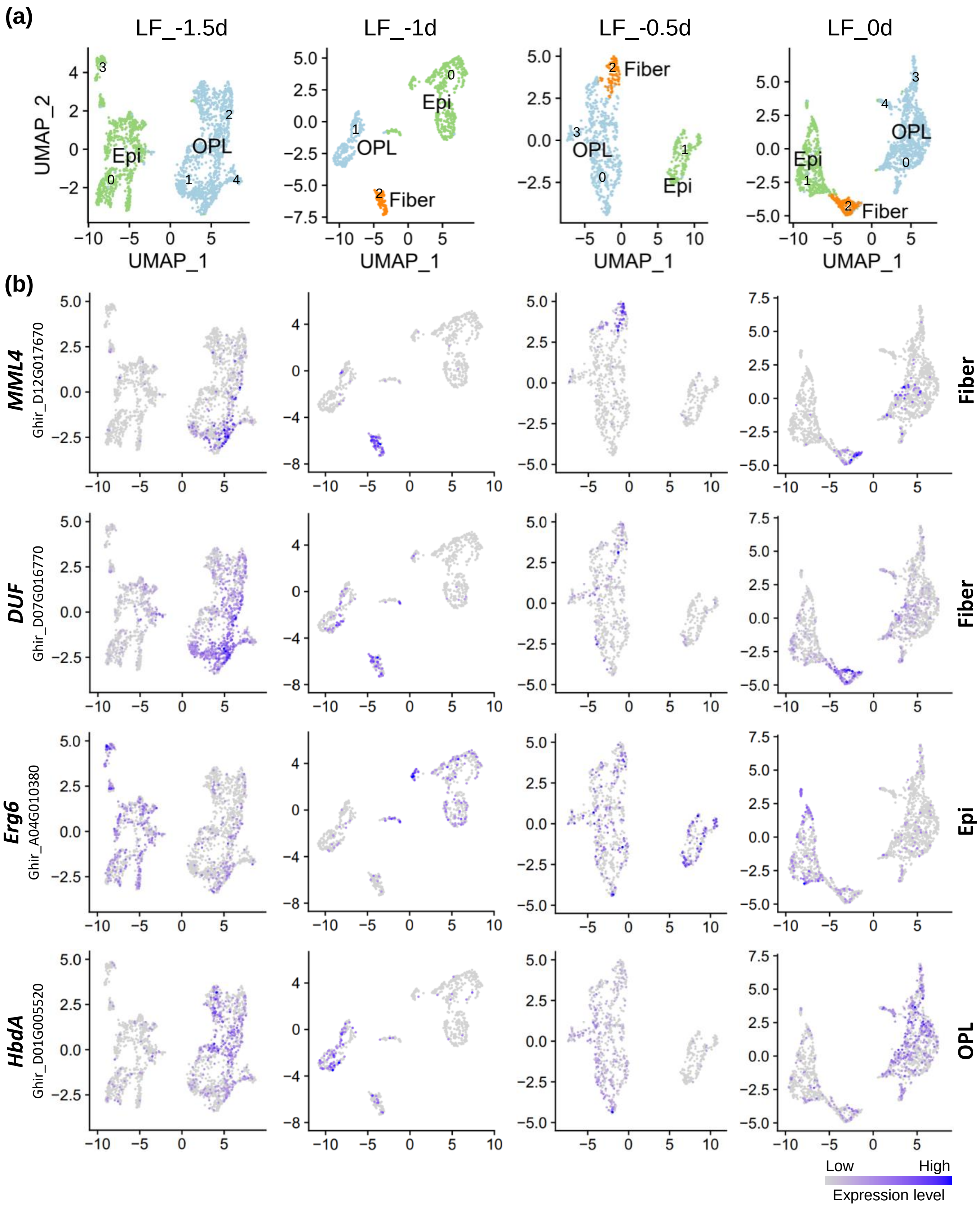

Fig.S8 Clustering and annotation of four Xu142_LF samples.

(a) UMAP projection plot showing major clusters of the single cell transcriptomes of Xu142_LF sample at -1.5, -1, -0.5 and 0 DPA, respectively. Each dot denotes a single cell. Colors denote corresponding clusters. Resolution was 0.2. **(b)** UMAP visualization of cell-type representative marker genes. *MML4* was a known fiber marker gene. *DUF*, *Erg6* and *HbdA* were proved to be marker gene of fiber, epidermis (Epi) and outer pigment layer (OPL), respectively (See Fig.S5).

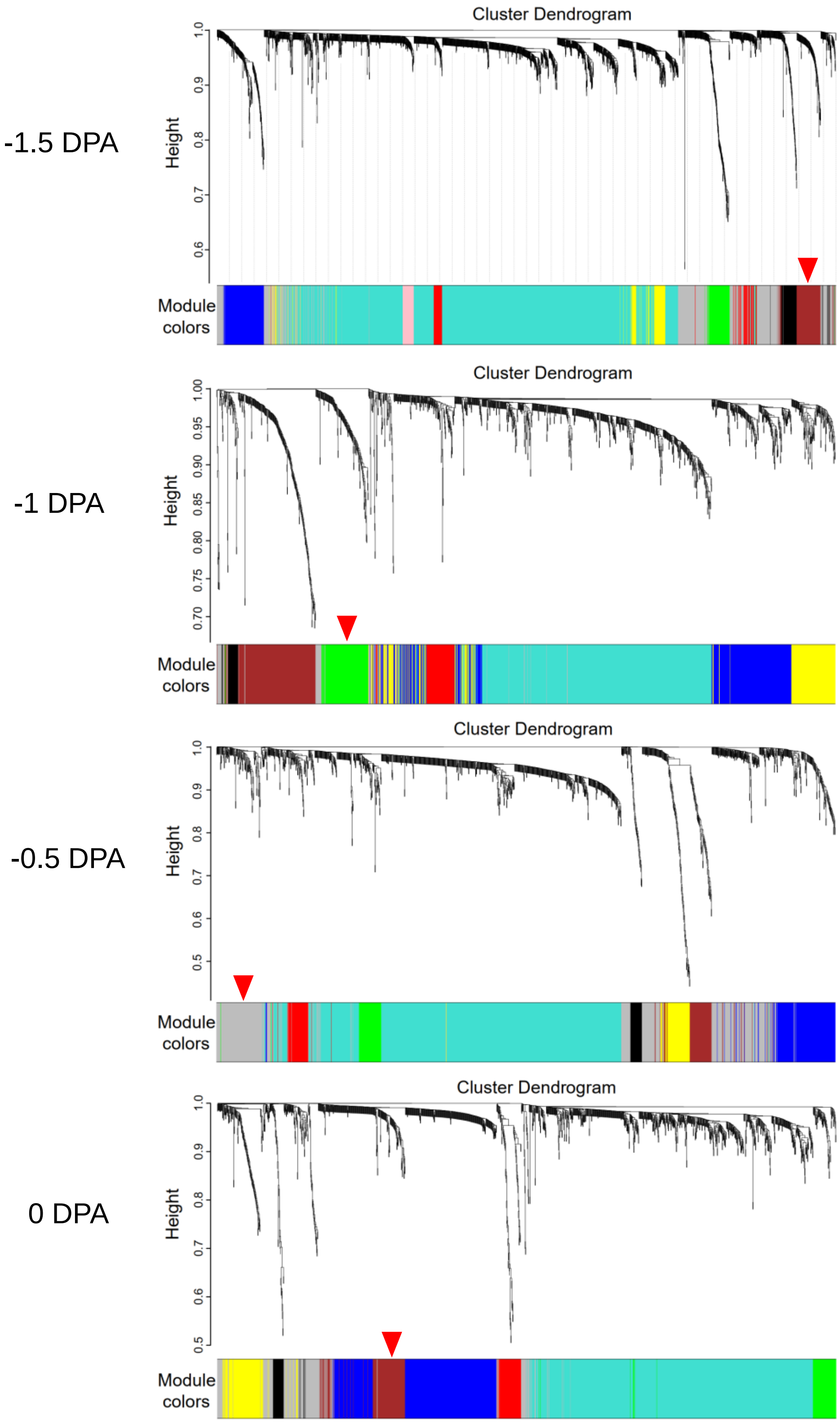

Fig.S9 Hierarchical cluster tree showing co-expression modules identified by WGCNA.

Samples of -1.5, -1, -0.5 and 0 DPA were calculated respectively. Each leaf in the tree is one gene. The major tree branches constitute several gene modules labeled by different colors. Red triangles point to the module containing genes enriched in fiber cluster (-1, -0.5 and 0 DPA) or cells that may be differentiated into fiber (-1.5 DPA). power = 4.

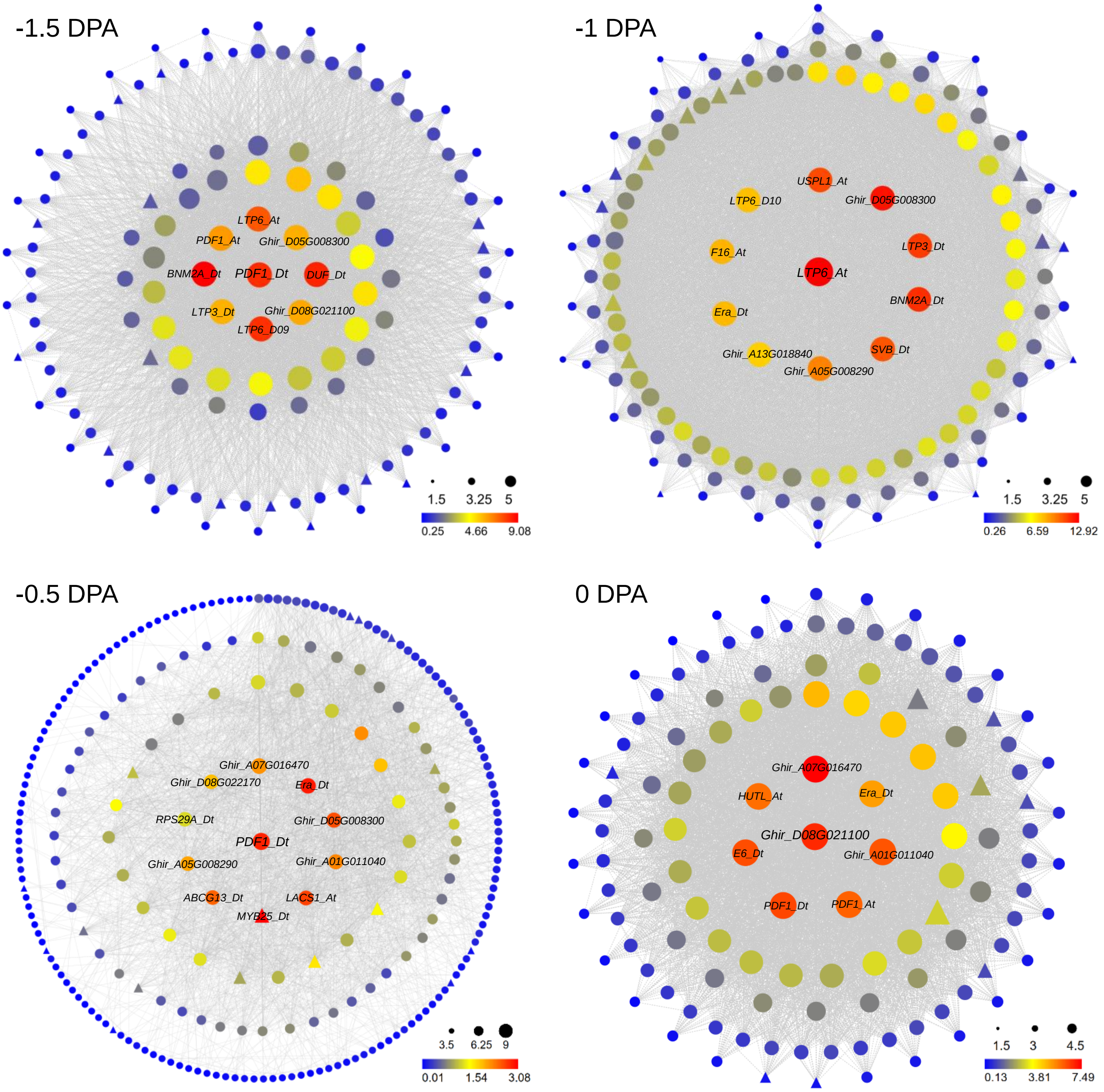

Fig.S10 Networks related to fiber cell initiation.

Co-expression network of hub regulating genes identified at -1.5, -1, -0.5 and 0 DPA were analyzed, respectively. Lines indicate edge weight (> 0.01) for each pair of genes. Each circle represents a gene, and triangle represents transcription factor. Node size is equivalent to the number of predicted connections. Node color represents the weight abundance of predicted connections.

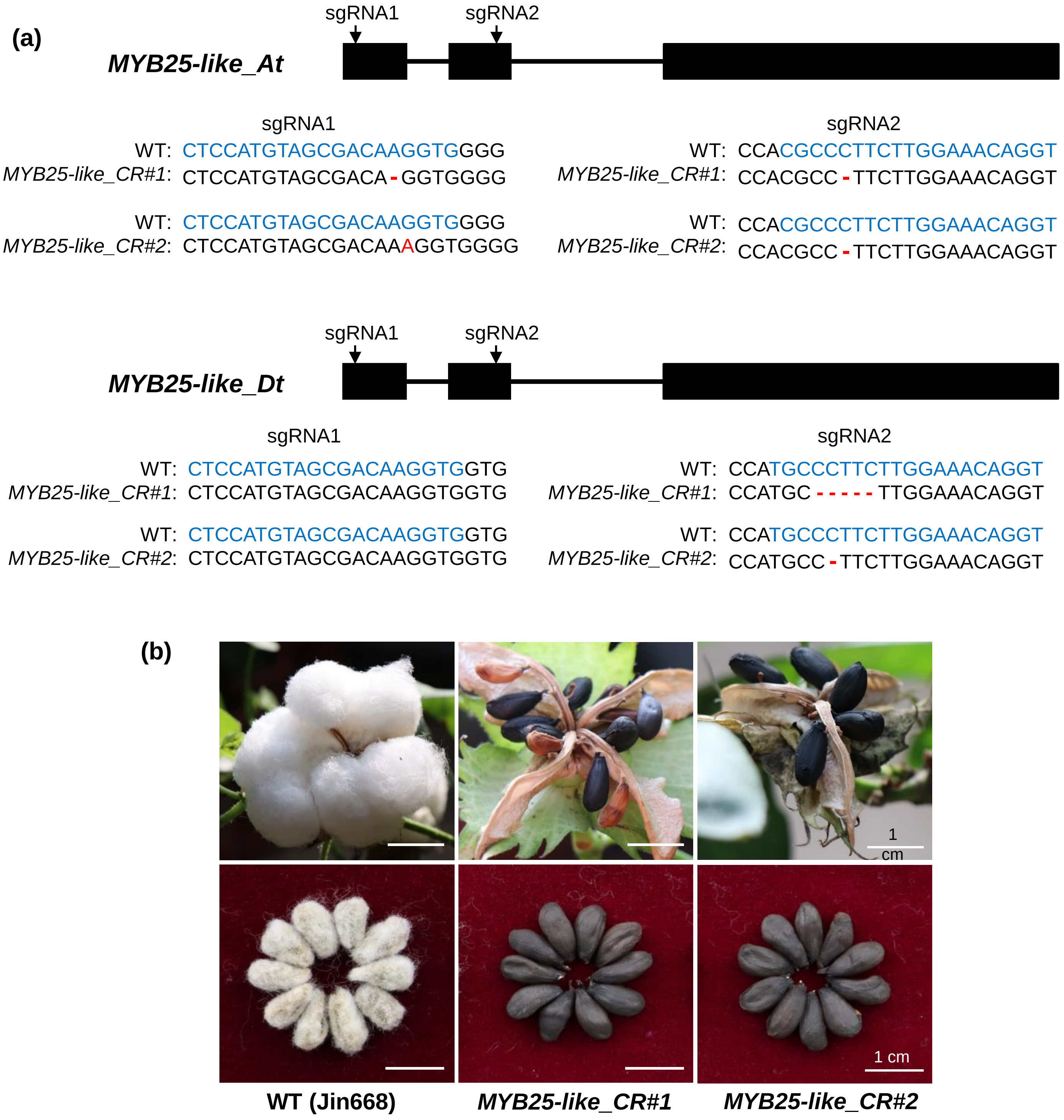

Fig.S11 Characterization of cotton CRISPR editing lines on *MYB25-like* genes.
(a) The target mutation site of *MYB25-like* genes T_1 plants edited by cotton CRISPR/Cas9 system. The sgRNA target sites are shown in blue. Nucleotide deletions or insertions are highlighted in red. **(b)** The phenotype of lint and fuzz between WT (Jin668, the transgenic receptor material) and two *MYB25-like_CR* lines. Scale bar, 1 cm.

(a)

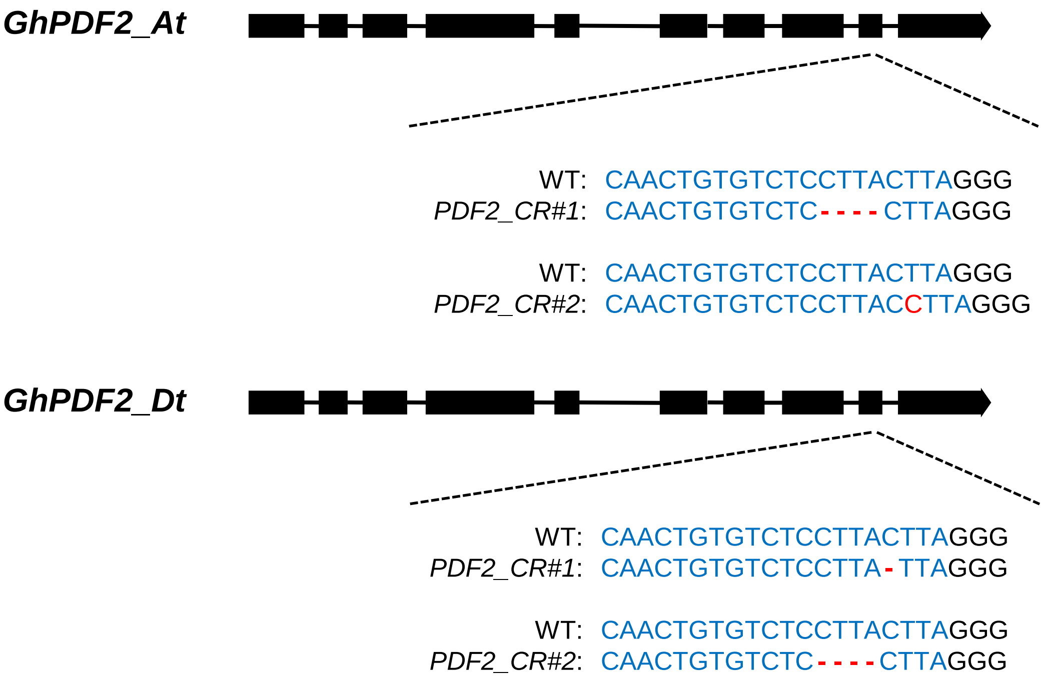

(b)

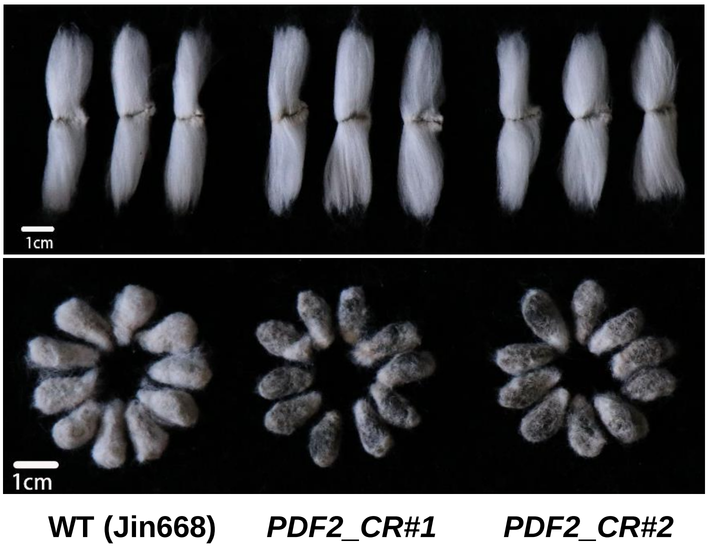

(c)

	UHML (mm)	Uniformity index (%)	Micronaire value	Strength (cN/tex)	Elongation (%)
WT	26.89 ± 0.82	85.77 ± 0.32	5.58 ± 0.13	28.30 ± 1.66	6.63 ± 0.06
PDF2_CR#1	27.12 ± 0.91	85.85 ± 0.21	5.37 ± 0.43	25.90 ± 0.85	6.55 ± 0.07
PDF2_CR#2	26.91 ± 0.60	87.50 ± 0.44 **	5.56 ± 0.08	26.30 ± 0.43	6.54 ± 0.05

Fig.S12 Characterization of cotton CRISPR editing lines on *PDF2* genes.
(a) The mutation site of T_1 plants with sgRNA target *PDF2* genes. The sgRNA target sites are shown in blue. **(b)** Lint and fuzz phenotype between WT and *PDF2_CR* lines. Scale bar, 1 cm. **(c)** Mature fiber quality between WT and *PDF2_CR* lines. **, $p < 0.01$.

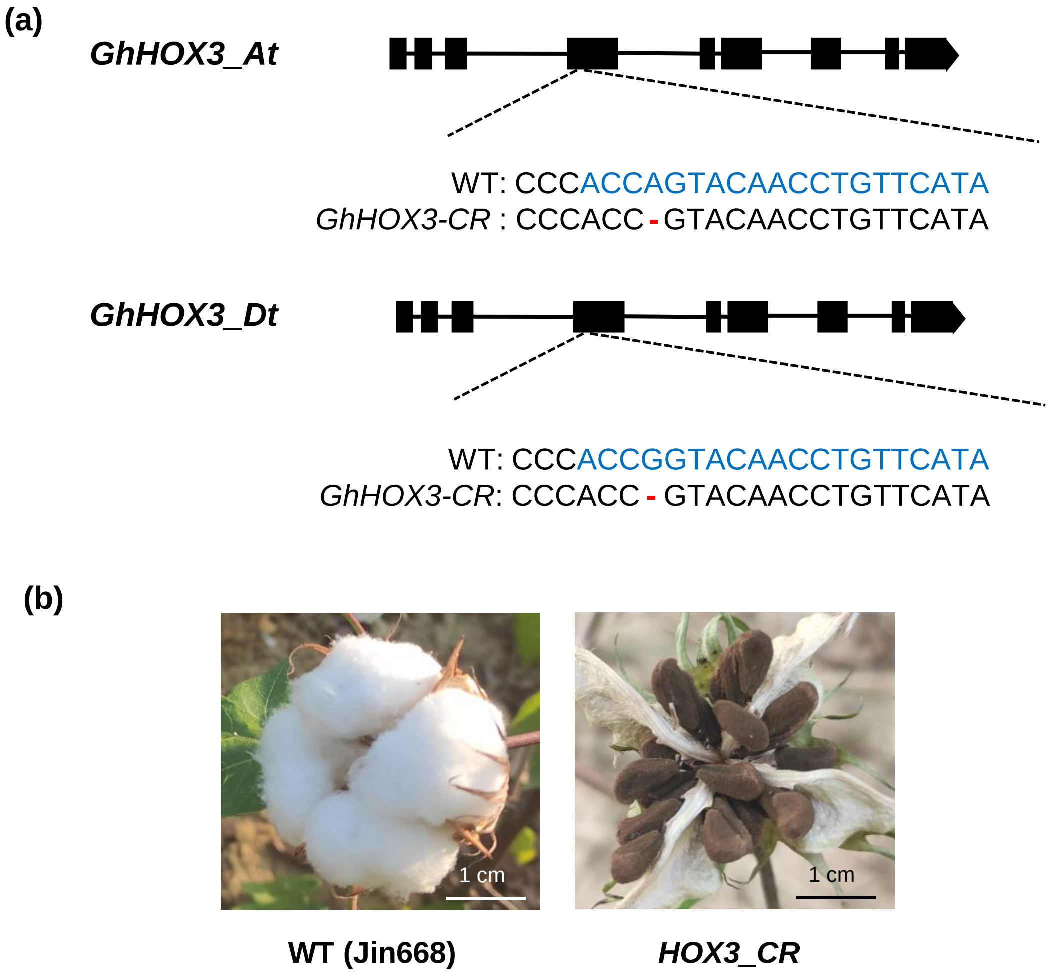

Fig.S13 Characterization of cotton CRISPR editing lines on *HOX3* genes.
(a) The mutation site of T_1 plants with sgRNA target *HOX3* genes. The sgRNA target sites are shown in blue. Nucleotide deletions are highlighted in red. **(b)** The phenotype of mature fiber between WT and *HOX3_CR* lines. Scale bar, 1 cm.
