# Supplementary material for: Single‐cell RNA‐seq reveals fate determination control of an individual fibre cell initiation in cotton (Gossypium hirsutum)
Source: Plant Biotechnol J. 2022 Oct 2;20(12):2372–88. doi: 10.1111/pbi.13918 (PMC9674311; doi:10.1111/pbi.13918)
Supplement: Supplementary file 14 — Appendix S1 Details of bioinformatics analysis. [file PBI-20-2372-s006.docx]

**Document S1. Details of bioinformatics analysis**

**Quality control**

We first used FastQC to perform basic statistics on the quality of the raw reads. Then, those read sequences produced by the Illumina pipeline in FASTQ format were pre-processed through Trimmomatic software, which can be summarized as below:

(1) Remove low-quality reads: scan the read with a 4-base wide sliding window, cutting when the average quality per base drops below 10 (SLIDINGWINDOW: 4:10)

(2) Remove trailing low quality or N bases (below quality 3) (TRAILING: 3)

(3) Remove adapters: there are two modes to remove the adapter sequence: a. alignment with the adapter sequence, the number of matching bases was greater than 7 and mismatch=2; b. when read1 and read2 overlapping base scoring greater than 30, removed non-overlapping portions (ILLUMINACLIP: adapter.fa:2: 30: 7)

(4) Drop reads below the 26 bases long

(5) Discard those reads that cannot form paired

The remaining reads that passed all the filtering steps were counted as clean reads and all subsequent analyses were based on this.

**Alignment, barcode assignment and UMI counting**

Cell Ranger 4.0.0 uses an aligner called STAR, which performs splicing-aware alignment of reads to the *Gossypium hirsutum* TM-1 reference genome (Wang et al., 2019) with version 1.1 of the annotation files. Cell Ranger then uses the transcript annotation GTF to bucket the reads into exonic, intronic, and intergenic. A read is exonic if at least 50% of it intersects an exon, intronic if it is non-exonic and intersects an intron, and intergenic otherwise. For reads that align to a single exonic locus but also align to 1 or more non-exonic loci, the exonic locus is prioritized and the read is considered to be confidently mapped to the exonic locus with MAPQ 255. Cell Ranger further aligns exonic reads to annotated transcripts, looking for compatibility. A read that is compatible with the exons of an annotated transcript, and aligned to the same strand, is considered mapped to the transcriptome. If the read is compatible with a single gene annotation, it is considered uniquely (confidently) mapped to the transcriptome. Only reads that are confidently mapped to the transcriptome are used for UMI counting. Run ‘cellranger count’ function with “-id, -transcriptome, -fastqs, -sample” arguments to implement the above process and generate single-cell gene counts. The clean data of each sample was processed according to the above function one by one. In result files, the gene-cell matrices, a folder named ‘filtered_feature_bc_matrix’ (containing 3 files: ‘barcodes.tsv’, ‘features.tsv’ and ‘matrix.mtx’), served for further analyses.

**PCA, UMAP and tSNE analysis**

The gene-cell matrices (take LF_0d sample as example: 3679 cells, 70199 genes) were load into the Seurat package (v. 4.0.1) with ‘Read10X’ function, which was implemented in R (v. 4.0.0). To remove doublets and no-load cells, we filtered the cells with ‘500 <= unique gene counts <= 5000’. To remove dead cells, we filtered the cells with mitochondrial sequence over 5%. The genes expressed in at least 10 single cells ware kept. After filtering, 35169 genes across 1703 cells were used for downstream analysis. For data normalization, two methods ‘LogNormalize’ and ‘CLR’ were performed respectively (Schneider et al., 2021). Through comparing the distributions on expression values, ‘CLR’ normalization result was chose for highly variable genes calculating with ‘FindVariableFeatures’ function. A total of 3000 highly variable genes were used for clustering analysis. Run the ‘RunPCA’ function to reduce the scaled data to 50 PCs (default is 50) depending on the 3000 genes. To determine the final number of PCs applicable for clustering, we evaluate the points where the percentage change between consecutive PCs less than 0.1%, and then 14 PCs were selected. For clusters identifying, run the ‘FindNeighbors’ function (“dims = 1:14”) first, then run ‘FindClusters’ function with “resolution = 0.8”. The data structures were separately visualized and explored by *t*-SNE (run the ‘RunTSNE’ function with “‘dims = 1:14’, ‘perplexity = 60’”) and UMAP (run the ‘RunUMAP’ function with “‘dims = 1:14’, ‘min.dist = 0.2’”). Cluster marker genes were identified using function ‘FindAllMarkers’ with “‘min.pct = 0.25’, ‘thresh.use = 0.25’, ‘logfc.threshold = 0.25’”. From the 3406 cluster-enriched genes, we selected the genes that enriched only in one cluster as cluster-specific marker genes. The filtration on the gene-cell matrices of other samples were performed as the same process above with related parameters adjusted.

**Pseudo-Time Analysis**

The Monocle (v. 2.16.0) package implemented in R (v. 4.0.0) was used to deeply analyze the process of cell differentiation. This analysis process was performed as Zhang et al. reported (Zhang et al., 2019) with a few parameters adjusted. Briefly, extract the subset of raw data, calculate gene’s expression variance (run ‘dispersionTable’) and choose variable genes (the ‘‘mean_expression’’ parameter), reduce the data’s dimensionality (run ‘reduceDimension’ with “max_components = 2, reduction_method = ‘DDRTree’”), describe the cell’s transition (run ‘orderCells’), plot the cell trajectory (run ‘plot_cell_trajectory’), analyze the branch-dependent genes (run ‘BEAM’) and visualize the significantly branch-dependent genes (run ‘plot_genes_branched_heatmap’).

**Function Method Description**

Gene Ontology (GO) enrichment analysis provides all GO terms that significantly enriched in selected genes comparing to the genome background. Firstly, all DEGs were mapped to GO terms in the Gene Ontology database (http://www.geneontology.org/), gene numbers were calculated for every term, significantly enriched GO terms in selected genes comparing to the genome background were defined by hypergeometric test. The calculated p-value was gone through FDR Correction, taking FDR ≤ 0.05 as a threshold. GO terms meeting this condition were defined as significantly enriched GO terms in selected genes. This analysis was able to recognize the main biological functions that selected genes exercise.

KEGG (Kanehisa and Goto, 2000) is a database resource for understanding high-level functions and utilities of the biological system, such as the cell, the organism and the ecosystem, from molecular-level information, especially large-scale molecular datasets generated by genome sequencing and other high-through put experimental technologies (http://www.genome.jp/kegg/). Pathway enrichment analysis identified significantly enriched metabolic pathways or signal transduction pathways in selected genes comparing with the whole genome background. Significantly enriched pathways in selected genes comparing to the genome background were defined by hypergeometric test. The calculated p-value was gone through FDR Correction, taking FDR ≤ 0.05 as a threshold. Pathways meeting this condition were defined as significantly enriched pathways in selected genes.

All these functional analyses described above was performed using the OmicShare tools, a free online platform for data analysis (www.omicshare.com/tools).

**References**

**Kanehisa, M., and Goto, S.** (2000). KEGG: Kyoto Encyclopedia of Genes and Genomes. Nucleic Acids Res. **28**: 27-30.

**Schneider, I., Cepela, J., Shetty, M., Wang, J., and Starr, T.K.** (2021). Use of "default" parameter settings when analyzing single cell RNA sequencing data using Seurat: a biologist's perspective. J Transl Genet Genom **5**: 37-49.

**Wang, M., Tu, L., Yuan, D., Zhu, D., Shen, C., Li, J., Liu, F., Pei, L., Wang, P., Zhao, G.*, et al.*** (2019). Reference genome sequences of two cultivated allotetraploid cottons, Gossypium hirsutum and Gossypium barbadense. Nat Genet **51**: 224-229.

**Zhang, T.Q., Xu, Z.G., Shang, G.D., and Wang, J.W.** (2019). A Single-Cell RNA Sequencing Profiles the Developmental Landscape of Arabidopsis Root. Mol Plant **12**: 648-660.
